# The optimal exercise modality and intensity for hemodialysis patients incorporating Bayesian network meta-analysis and systematic review

**DOI:** 10.3389/fphys.2022.945465

**Published:** 2022-09-19

**Authors:** Yangyang Song, Lei Chen, Meng Wang, Quan He, Jinhong Xue, Hongli Jiang

**Affiliations:** Dialysis Department of Nephrology Hospital, The First Affiliated Hospital of Xi’an Jiaotong University, Xi’an, China

**Keywords:** hemodialysis, blood pressure control, network meta-analysis, dialysis efficiency, exercise dosage

## Abstract

**Background:** Physical inactivity is highly prevalent in patients with hemodialysis, and a large body of evidence reported the positive effect of different exercise modalities on their health outcomes. However, the effective dosage of exercise for hemodialysis patients still requires verification.

**Objective:** We aimed to determine the most effective exercise intensity and modality for improvements in physical function, blood pressure control, dialysis adequacy, and health-related quality of life for hemodialysis patients.

**Design:** Systematic review with network meta-analysis of randomized trials.

**Data sources:** Five electronic databases (PubMed, EMBASE, Web of Science, Cochrane CENTRAL, and Scopus) were searched for randomized controlled trials. Data extraction and quality appraisal were conducted by two authors independently. Data were analyzed by the R (version.3.6.2) and the Stata (version.15.0).

**Result:** We included 1893 patients involving four exercise modalities and six exercise intensities. Combined training (aerobic exercise plus resistance exercise) has been the top-ranking exercise modality for improving the 6-min walk test (6MWT) (surface under the cumulative ranking curve analysis (SUCRA) score, 90.63), systolic blood pressure control (SUCRA score, 77.35), and diastolic pressure control (SUCRA score, 90.56). Moreover, the top-ranking exercise intensity was moderate–vigorous for 6MWT (SUCRA score, 82.36), systolic blood pressure (SUCRA score, 77.43), and diastolic blood pressure (SUCRA score, 83.75). Regarding dialysis adequacy and health-related quality of life, we found no exercise modality or intensity superior to the placebo.

**Conclusion:** This network meta-analysis indicated that combined training and moderate–vigorous intensity might be the most effective interventions to improve 6MWT and blood pressure control. This finding helps further guide clinical exercise prescriptions for hemodialysis patients.

**Systematic Review Registration**: [https://www.crd.york.ac.uk/PROSPERO/], identifier [CRD42021268535].

## 1 Introduction

Chronic kidney disease (CKD) is one of the most prevalent global health problems (Collaboration, 2020). The number of CKD patients transitioning to maintenance hemodialysis (HD) has increased in recent years (Yang et al., 2021). Nearly 25 million patients required dialysis therapy in 2020, which is expected to double by 2030 (Liyanage et al., 2015). Hemodialysis has been considered a standard alternative treatment for patients with kidney failure (O’Hare et al., 2003), but it comes along with a higher prevalence of cardiovascular diseases (Ahmadmehrabi and Tang, 2018) and mortality (Robinson et al., 2014). Regular exercise among HD patients was associated with lower mortality risk (Tentori et al., 2010). However, HD patients usually choose a sedentary lifestyle (Stack and Murthy, 2008). The Dialysis Outcomes and Practice Patterns Study (DOPPS), which consisted of 20,920 HD participants in 12 countries, showed that 43.9% never exercised and only 5.7% exercised four to five times per week (Tentori et al., 2010).

Many physiological factors contribute to physical inactivity in HD patients. First, over 80% of HD patients had chronic obstructive pulmonary diseases (Plesner et al., 2016), and cardiovascular diseases (CVDs) exist in nearly 50% of HD patients (Ahmadmehrabi and Tang, 2018). Since cardiac and pulmonary dysfunction significantly reduces absolute ventilation, HD patients can hardly increase ventilation in response to activity (Fedeli et al., 2017; Rangaswami et al., 2019; McGuire et al., 2020). Second, the prevalence of sarcopenia among HD patients was up to 65% (D’alessandro et al., 2018). HD-related sarcopenia was associated with reduced exercise capacity (Hirai et al., 2016; Kirkman et al., 2021). Third, malnutrition, anemia, and iron deficiencies were common complications of HD patients, lowering the oxygen-carrying and aerobic capacity (Hannan and Bronas, 2017; Macdougall, 2017). Other factors related to HD patients’ exercise intolerance include fear of injuries, symptoms of debilitation, and fatigue (Delgado and Johansen, 2012; Wang et al., 2020).

Previous evidence demonstrated that exercise could improve cardiovascular function, physical function, and quality of life and relieve restless legs syndrome, muscle cramping, and fatigue for HD patients (Ferrari et al., 2020; Hargrove et al., 2021). Most systematic reviews have compared the control group with different exercise modalities (e.g., aerobic training, resistance training, and aerobic exercise plus resistance training) on physical function, cardiovascular health, and hemodialysis efficiency (Sheng et al., 2014; Ferreira et al., 2019; Ferrari et al., 2020). Some systematic reviews showed that resistance training (RT) and electrostimulation (EMS) could improve the 6-min walk distance (Ferrari et al., 2020). The 6MWT was one of the most commonly used tools for evaluating exercise capacity and endurance (Vogiatzaki et al., 2022). The change of 6MWT was also considered a sensitive indicator for assessing the effectiveness of an exercise intervention on hemodialysis patients (Kono et al., 2014). Aerobic training (AT) can improve HD efficiency (Ferreira et al., 2019). Another systematic review demonstrated that RT and combined training (CT: aerobic exercise plus resistance training) has no effect on 6MWT, only AT can improve 6MWT, and exercise has no effect on blood pressure control (Huang et al., 2019). Although these results have verified the effectiveness of exercise among HD patients, they are controversial. Few studies have compared the effect of different modalities on HD patients. Only one network meta-analysis (Scapini et al., 2019) compared the effect of three exercise modalities simultaneously, but the heterogeneity was high in this systematic review. As an attractive strategy, EMS was less investigated. We have no idea which specific exercise modality is appropriate for patients.

There is also a dose–response relationship between exercise intensity and health outcomes for HD patients. First, some trials showed that compared to those with low exercise intensity, higher intensity could contribute to improvements in cardiovascular outcomes among hemodialysis patients (Tzvetanov et al., 2014; Greenwood et al., 2015; Chan et al., 2018). Some trials showed that moderate and vigorous exercise could improve aerobic capacity and health-related quality of life and lower blood pressure (Reboredo et al., 2011). On the other hand, “morphologic muscle threshold” might explain the phenomenon that exercise does not affect hemodialysis efficiency (Parsons et al., 2006; Pellizzaro et al., 2013). Exercise must exceed a certain threshold to increase enough muscle blood flow and enlarge the capillary surface and then contribute to circulating toxins transferred to the intravascular compartment and removed by dialysis (Brown et al., 2018; Andrade et al., 2019). However, no systematic review or meta-analysis is available to compare the effect of various training intensities on HD patients’ health outcomes.

Due to variations in evidence and limited guidelines, it was an ongoing challenge for dialysis clinicians to support structured exercise programs as routine care (Salhab et al., 2019). Most dialysis physicians (85%) and nurses (83.3%) had no experience with interventional exercise programs for HD patients (Michou et al., 2019). The development of exercise programs has been largely overlooked for HD patients (Regolisti et al., 2018; Lambert et al., 2022) and falls far behind that of other chronic diseases (obesity, diabetes, stroke, and hypertension), for which the consensus for exercise prescription or guideline had already been established (Hansen et al., 2018; Kim et al., 2019; Teich et al., 2019; Alpsoy, 2020).

To our knowledge, there was little evidence about the optimal exercise modality and intensity for HD patients. Specific exercise prescriptions for hemodialysis patients should be designed by physical therapists and facilitate physical exercise for HD patients. To address the knowledge gap, we conducted a Bayesian network meta-analysis combining all available direct and indirect evidence across trials to compare the effect of various exercise modalities and intensities. Our research question for this systematic review was as follows: which specific exercise modality and intensity is the optimal exercise intervention to improve physical capacity, dialysis efficiency, blood pressure control, and health-related quality of life?

## 2 Methods

### 2.1 Literature search and selection criteria

The network meta-analysis was conducted in agreement with the PRISMA network meta-analysis (PRISMA-NMA) (Hutton et al., 2016), and it has been registered in the PROSPERO database (registration number ID: CRD42021268535).

We searched PubMed, EMBASE, Web of Science, Cochrane CENTRAL, and Scopus (from their inception date to 12 June 2022). The following keywords or combinations were used: exercise, aerobic exercise, resistance exercise, electrostimulation, hemodialysis, and chronic kidney disease. The complete search strategy used in PubMed is shown in Supplementary Appendix S1. Trials were also included by manually searching the reference lists of relevant reviews. We have no restrictions on the language of the publications.

We screened all included RCTs according to the criteria of the PICOS (participants, interventions, comparators, outcomes, and study design). The population group of interest was adults (≥18 years) with a chronic renal disease requiring hemodialysis. Interventions are exercise training such as aerobic training (AT), resistance training (RT), combined training (CT), and electrostimulation (EMS). The control group means the non-exercise training group. We included studies that have at least one of the outcome measures: physical function (6-min walk distance) and blood pressure (systolic blood pressure and diastolic blood pressure). The 6MWT is inexpensive, safe, and easier to perform and repeat, even though it is less sensitive to detect physical performance changes than the cardiopulmonary exercise test (Galie et al., 2016). The American Thoracic Society guideline advised that (Uszko-Lencer et al., 2017) the 6MWT was the gold standard tool to assess functional exercise capacity and activities of daily living, and moreover, it is better tolerated for chronic diseases than other walk tests (such as some tests needed to reach a speed). The 6MWT has been widely validated to assess exercise capacity in kidney diseases, including end-stage kidney disease (Shi et al., 2017), kidney transplant candidates (Cheng et al., 2020), and non-kidney solid organ transplantation (Kobashigawa et al., 2019). A long cohort also showed that compared to other physical performance assessment tools, only 6MWT was a significant predictor of severe mortality for hemodialysis patients (Vanden Wyngaert et al., 2022). Blood pressures were measured when patients were at rest. HD efficiency was measured using single-pool Kt/V (Churchill and Patri, 2021), the most common measurement for dialysis adequacy worldwide. “K” represents dialyzer urea clearance, “t” means the duration time of a single dialysis session, and “V” is the volume of urea distribution that is equal to total body water (Gotch et al., 2000). The Kidney Disease Outcomes Quality Initiative (KDOQI) guidelines recommended that single-pool Kt/V should be 1.2 or higher (Daugirdas et al., 2015). Moreover, mental- or physical health-related quality of life was measured by the short-form 36 health questionnaire (SF-36), which consisted of physical and mental component dimensions (Kraus et al., 2016). Physical health consisted of four scales (physical function, role physical, bodily pain, and general health), and mental health included four scales (vitality, social functioning, role-emotional, and mental health), and higher scores mean better health status (Turkmen et al., 2012).

### 2.2 Data abstraction

Two authors extracted data from the included trials (Yangyang Song and Lei Chen). The following information was extracted: year of publication, basic characteristics of patients, and details of intervention (exercise modality, intensity, frequency, and duration). The same two authors independently used the Cochrane Collaboration tool (Sterne et al., 2019) to assess the quality of each included trial and included randomized sequence generation, allocation concealment, performance bias, detection bias, incomplete outcome data, selective reporting, and other biases. Any disagreements were resolved by discussion with the third author (Hongli Jiang).

The trials with AT, RT, and CT were summarized into five intensities: light, light–moderate, moderate, moderate–vigorous, and vigorous, according to ACSM recommendations (Garber et al., 2011; Scharhag-Rosenberger et al., 2015). The indicators of exercise intensity are HR (heart rate), HRR (heart rate reserve), and VO_2max_ or some subjective parameters such as the rating of perceived exertion (RPE). The resistance exercise was classified by repetition maximum (RM). We added an intensity according to the patient’s need, which means there was no specific exercise velocity or target heart rate for patients to achieve (Molsted et al., 2004; Pomidori et al., 2016). The classification details of the intensity of each exercise are shown in Supplementary Appendix S2 eTable1. The summarization process was performed by two authors independently. We contacted the authors when the data were unavailable.

### 2.3 Synthesis analysis

We performed a pairwise meta-analysis using a random-effect model. The mean difference (MD) and a 95% confidence interval (CI) were used as effect estimates among different studies. Heterogeneity was assessed by I^2^ statistics, 0–40%, 40%–60%, 60%–75%, and 75–100%, which indicated low, moderate, substantial, and considerable heterogeneity, respectively (Hutton et al., 2016). The unit-of-analysis error may exist in studies with two or more experimental groups. To overcome this error, we split these trials into two or more groups, with one control group in the pairwise meta-analysis (Andreato et al., 2019).

We performed NMA using a Bayesian framework noninformatively prior to distributions and the Markov chain Monte Carlo method (MCMC). The Bayesian network meta-analysis can provide evidence from direct and indirect comparisons. The model allows comparisons of the different treatments simultaneously. The network plot was generated to represent the connection between each treatment. The lines in the plot represented direct comparisons between two treatments, the width of the lines represented the number of trials, and the size of each node represented the number of participants (Salanti et al., 2011). We ranked the treatments using the surface under the cumulative ranking curve (SUCRA). SUCRA reported the cumulative ranking probabilities and considered the more precise estimation of ranking probabilities (Mbuagbaw et al., 2017). The value of SUCRA close to 1 represented the best intervention (Salanti et al., 2011). We presented the treatment effect and the corresponding 95% CI by league tables.

For each outcome, heterogeneity was evaluated using the I^2^ statistic. The node-splitting approach was used to examine whether there exists any inconsistency between direct and indirect evidence, and the *p*-value was higher than 0.05, which indicated no significant inconsistency. We would analyze using clinical factors if there does exist statistical inconsistency and heterogeneity. Sensitive analysis was performed by excluding the high risk of trials. We also judged publication bias by inspecting the asymmetry of a comparison-adjusted funnel plot and the *p*-value of Egger^’^s test. Serial analyses were performed in STATA (Stata version 15.0), R language, and the “RJAGS” package.

Our analyses were based on previously published trials, and there is no need for ethical approval and patient consent.

## 3 Results

### 3.1 Flow of studies through the review

**FIGURE 1:**
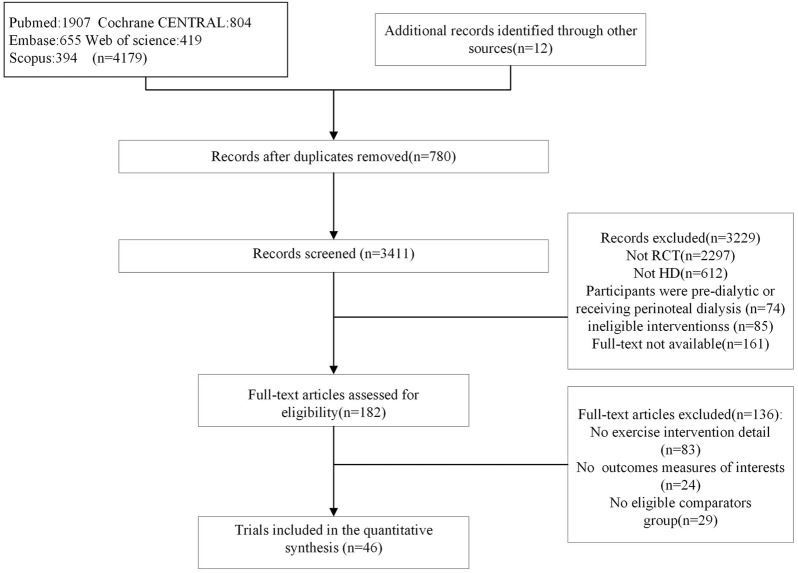
Flow of studies through the review.

The flow diagram of the included studies is presented in Figure 1. The initial search yielded 4,179 records, and 12 studies were included in the reference lists of published systematic reviews. A total of 780 duplicates were excluded, and 3,411 studies were excluded after abstract screening. A total of 182 studies were retrieved in full text for further consideration. In total, 46 trials were finally included in the qualitative analysis.

### 3.2 Study characteristics and risk of bias assessment

The 46 studies comprised 1893 participants, and the study duration ranged from 8 to 40 weeks. Among the studies included, 1,011 were allocated to exercise training interventions, and 882 were allocated to the non-exercise group. Exercise training interventions included aerobic (*n* = 417), resistance (*n* = 282), and combined aerobic and resistance exercise (*n* = 252), EMS (*n* = 60).

Nineteen studies compared the effects of AT and the control group (Painter et al., 2002; Tsuyuki et al., 2003; Sakkas et al., 2008; Toussaint et al., 2008; Koh et al., 2010; Reboredo Mde et al., 2010; Wilund et al., 2010; Giannaki et al., 2013; Mohseni et al., 2013; Wu et al., 2014; Groussard et al., 2015; Liao et al., 2016; Pomidori et al., 2016; Cooke et al., 2018; Fernandes et al., 2019; Lin et al., 2021; Perez-Dominguez et al., 2021; Kim et al., 2022; Vogiatzaki et al., 2022). Nine studies evaluated RT and the control group. (Cheema et al., 2007; Pellizzaro et al., 2013; Kirkman et al., 2014; Abreu et al., 2017; Rosa et al., 2018; Dong et al., 2019; Martins do Valle et al., 2020; Gadelha et al., 2021).

Ten studies evaluated CT (DePaul et al., 2002; Molsted et al., 2004; van Vilsteren et al., 2005; Ouzouni et al., 2009; Frih et al., 2017; Huang et al., 2019; Hatef et al., 2020; Yeh et al., 2020; Assawasaksakul et al., 2021; Myers et al., 2021), and three studies evaluated EMS (Roxo et al., 2016; Schardong et al., 2017; Suzuki et al., 2018). In total, 35 studies were two-arm studies, three were three-arm studies (Afshar et al., 2010; Dobsak et al., 2012; McGregor et al., 2018), and two trials were four-arm studies (Kopple et al., 2007; Thompson et al., 2016). The duration of most trials ranged from 8 weeks to 12 months. The exercise duration was 8–12 weeks in 26 trials, 12–24 weeks in 11 trials, and ≥24 weeks in nine trials. The frequency of exercise training per week ranged from 2 to 4 times except for one study, which was performed once per day (Myers et al., 2021). The exercise duration for most trials per session was 30–90 min. The detailed characteristics of included trials are presented in Supplementary Appendix S3 eTable2.

The risk of bias assessment for each study is shown in Supplementary Appendix S4. Most trials are not of high methodological quality. As the included studies involved exercise interventions, it may be difficult to blind patients and investigators, and most trials tend to have unclear risks of performance bias, and eight studies have high risks of detection bias. Random sequence generation was reported in only 20 studies (43.48%), and 26 studies (56.52%) had unclear risks. Allocation concealment in 13 studies (28.26%) was reported, while 32 studies had unclear bias (69.57%). Nearly half of the studies did not report incomplete outcome data (50%). Only ten trials exhibited a low risk of selective outcome reporting (21.73%), and 33 studies (71.74%) had an unclear bias. Twenty studies (43.48%) were judged high or unclear in the domain risk of other bias since they did not report the sample size calculation. It was challenging to explain the result, especially in some studies with small sample sizes.

### 3.3 Pairwise meta-analysis

The detailed results of the pairwise meta-analysis are shown in Supplementary Appendix S5 eTable3. For 6MWT, CT (MD = 4.9, 95% CI = 3.1 to 6.7, I^2^ = 0%), AT (MD = 3.3, 95% CI = 0.7 to 6.0, I^2^ = 0%), and RT (MD = 2.5, 95% CI = 0.56 to 4.5, I^2^ = 0%) reached statistical significance than the control group. However, EMS had limited effect on 6MWT (MD = 2.3, 95% CI = −2.1 to 6.6, I^2^ = 0%). Moderate–vigorous intensity (MD = 4.5, 95% CI = 1.9 to 7.1, I^2^ = 4.8%) is superior in the control group. Moderate (MD = 5.0, 95% CI = 1.80 to 7.7, I^2^ = 0%) and moderate–vigorous intensity (MD = 4.6, 95% CI = 0.80 to 8.4, I^2^ = 0%) are more efficient than light intensity. Regarding Kt/V, no exercise modality and intensity has got statistical significance improvement than the control group.

As for systolic and diastolic blood pressure, no exercise modality significantly reduced systolic or diastolic blood pressure more than the control group. Moderate–vigorous exercise significantly reduced systolic blood pressure (MD = −8.7, 95% CI = −17 to −1.6, I^2^ = 70.8%) and diastolic blood pressure (MD = −4.9, 95% CI = −9.9 to −0.35, I^2^ = 74.2%) than the control group.

For mental health-related quality of life, CT (MD = 3.6, 95% CI = −4.1 to 11.0, I^2^ = 58.8%), AT (MD = 3.8, 95% CI = −2.2 to 10.0, I^2^ = 60.0%), and RT (MD = 5.7, 95% CI = −5.4 to 17.0, I^2^ = 70.3%) did not reach statistical significance than the control group. Moderate (MD = 3.6, 95%CI = −7.6 to 16.0, I^2^ = 84.0%) and moderate–vigorous intensity (MD = 2.4, 95% CI = −4.6 to 9.4, I^2^ = 66.6%) is not superior to that in the control group. For physical health-related quality of life, CT (MD = 3.6, 95% CI = −5.9 to 13.0, I^2^ = 0%), AT (MD = 6.9, 95% CI = −23.0 to 11.0, I^2^ = 77.4%), and RT (MD = 8.7, 95% CI = −3.0 to 21.0, I^2^ = 70.3%) did not reach statistical significance than the control group. Moderate (MD = −0.52, 95% CI = −12.0 to 11.0, I^2^ = 0%) and moderate–vigorous intensity (MD = 5.8, 95% CI = −1.2 to 13.0, I^2^ = 82.9%) is not superior to that in the control group. However, only one trial (Dobsak et al., 2012) compared the effect of intra-dialytic EMS (20 weeks) on health-related quality of life among HD patients, and significant improvement was observed in the mental function (*p* = 0.001) and physical function (*p* = 0.006) compared to that in the control group.

### 3.4 Synthesis results of network meta-analysis

**FIGURE 2:**
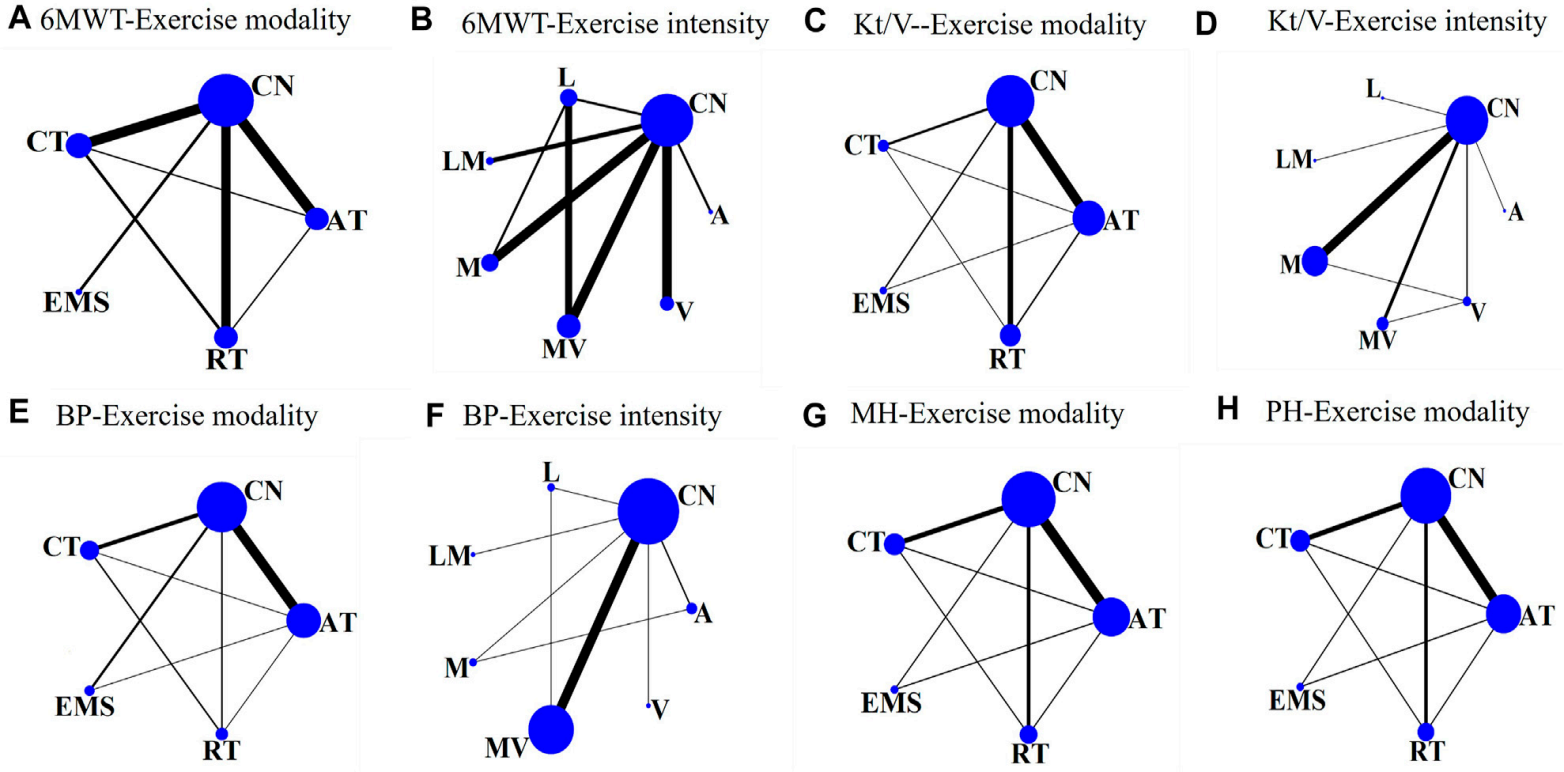
Network plots of clinical trials for hemodialysis patients comparing different exercise modalities or intensities to **(A)**: 6MWT-exercise modalities. **(B)**: 6MWT-exercise intensities. **(C)** Kt/V- exercise modalities. **(D)**: Kt/V-exercise intensities. **(E)** blood pressure-exercise modalities. **(F)**: blood pressure-exercise intensities. **(G)**: Mental health-exercise modalities. **(H)** Physical health-exercise modalities.

**FIGURE 3:**
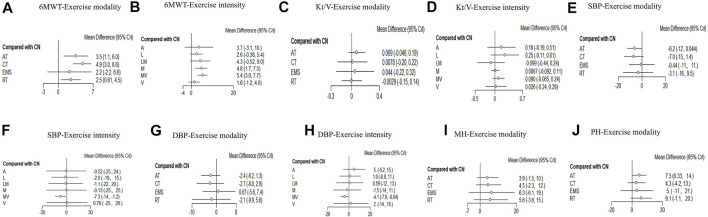
Forest plots for each outcome category represent the comparisons of the active intervention with no intervention (control group). **(A)**: 6MWT-exercise modalities and **(B)**: 6MWT-exercise intensities. **(C)** Kt/V-exercise modalities. **(D)**: Kt/V-exercise intensities. **(E)** Systolic blood pressure-exercise modalities. **(F)**: Systolic blood pressure-exercise intensities. **(G)**: Diastolic blood pressure-exercise modalities. **(H)**: Diastolic blood pressure-exercise intensities. **(I)** Mental health-exercise modalities. **(J)** Physical health-exercise modalities.

**FIGURE 4:**
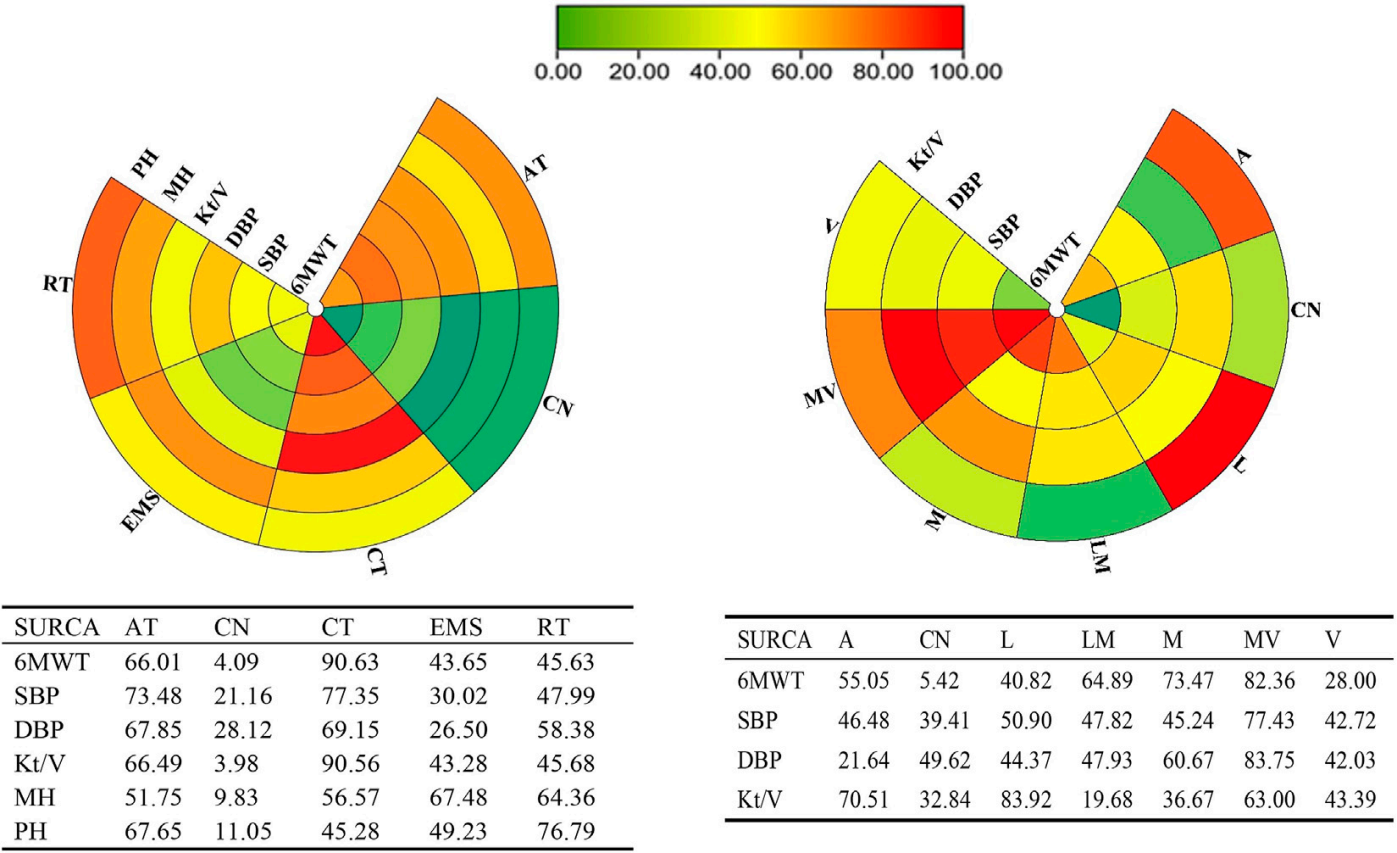
Schematic details of the treatments according to the surface under the cumulative ranking curve analysis (SUCRA).

Our network analysis of exercise modality was conducted among the four treatments. EMS was difficult to classify based on intensity. Only the studies on AT, RT, or CT were included to estimate the effect of different intensities. The visual network plot was performed to display evidence among different exercise modalities and exercise intensities. All network plots are presented in Figure 2. Each node represented a unique intervention or control group. The lines showed a direct relationship between interventions, and the width was weighted according to the number of trials between them. Figure 3 presents the interventions of mean difference and the 95% credible interval (CrI) in accordance with the control group. Supplementary Appendix S6 illustrates the effect sizes (MD) for all exercise modalities or intensities. Figure 4 shows the corresponding surface under the cumulative ranking curve analysis (SUCRA) in terms of different outcomes.

#### 3.4.1 Assessment of heterogeneity and inconsistency for each outcome

There was no significant statistical heterogeneity (I^2^<10%) for each exercise modality and intensity; more details are shown in Supplementary Appendix S7 eTable4. The assessment of incoherence global results for each outcome is presented in Supplementary Appendix S8 eTable5. The node split model results indicated no difference between direct and indirect outcomes (*p* values >0.05). The node-splitting results are presented in Supplementary Appendix S8 eTable6.

#### 3.4.2 Network meta-analysis results for 6MWT

Twenty studies that assessed the effect of different exercise modalities on 6MWT were eligible for network analysis. A total of five unique treatments were included in the network plot (Figure 2A). CT (MD = 4.89; 95% CI, 3.02–6.60), RT (MD = 2.50; 95% CI, 0.61–4.48), and AT (MD = 3.53; 95% CI, 1.09–6.01) were more effective than those of the control group (Figure 3A). EMS (MD = 2.23; 95% CI, −2.15–6.61) had no significant effect on 6MWT than in the control group. The result showed that CT is the best treatment with the highest probability (SUCRA score, 90.63).

A total of six unique exercise intensities were included in the network plot (Figure 2B). The light intensity (MD = 2.55; 95% CI, −0.52–5.39), light–moderate intensity (MD = 4.30; 95%CI, −0.53–9.01), and “A” (the intensity was according to the patient’s need) had no significant effect on 6MWT than in the control group. The moderate–vigorous intensity (MD = 5.36; 95% CI, 2.99–7.73) showed significantly superior efficacy over the control group (Figure 3B). The moderate–vigorous intensity was more efficient than the light intensity (MD = 2.82; 95% CI, 0.02–5.80) and the vigorous intensity (MD = 3.82; 95% CI, 0.39–7.44). The top-ranked interventions for 6MWT were moderate–vigorous intensity (SUCRA score, 82.36).

#### 3.4.3 Network meta-analysis results for hemodialysis efficiency

As for Kt/V, the network plot (Figure 2C) consisted of 21 studies with five different exercise modalities. No difference has been found between the intervention and control groups (Figure 3C). CT had the highest probability (SUCRA score, 90.56) of being the best treatment for this outcome. Twenty studies assessed the effect of seven different exercise intensities for Kt/V. The network plot is shown in Figure 2D. All intensities had a 95% CrI including the zero effect (Figure 3D). Light-intensity exercise was the most effective treatment (SUCRA score, 83.92).

To evaluate the effect of intradialytic exercise on improving Kt/V, we also conducted an analysis that only included studies in which the intervention was intra-dialytic exercise. Twenty studies were included in the analysis. The results showed that even intra-dialytic exercise exhibited no significant effect on improving Kt/V. The details of forest plots are presented in Supplementary Appendix S9. AT had the highest probability (SUCRA score, 74.14) and was the best treatment for this outcome. Light-intensity exercise was the most effective treatment (SUCRA score, 83.93).

#### 3.4.4 Network meta-analysis results for blood pressure

In the network meta-analysis of blood pressure, 21 studies on exercise modality’s effects and nineteen studies on different exercise intensities were included. Only two network plots of exercise modality (Figure 2E) and exercise intensity (Figure 2F) were presented for blood pressure because all studies simultaneously measured both systolic and diastolic blood pressure.

In the network meta-analysis of systolic blood pressure, none of the exercise training modalities was more effective than the control group (Figure 3E). Moderate–vigorous exercise intensity (MD = −7.33, 95% CI, −14.08, 1.25) was more effective in reducing systolic blood pressure than in the control group (Figure 3F). CT had the highest probability (SUCRA score, 77.35) and was the best treatment for systolic pressure control. Moderate–vigorous exercise intensity (SUCRA score, 77.43) has the best intensity for systolic pressure control.

In the network meta-analysis of diastolic blood pressure, all exercise modalities were statistically equivalent to the control group (Figure 3G). CT had the probability (SUCRA score, 69.15) to be the best treatment. Moderate–vigorous exercise intensity (MD = −4.13, 95% CI, -7.93, −0.64) was more effective in reducing systolic blood pressure than in the control group. The other exercise intensities had a 95% CrI, including the zero effect (Figure 3H). The moderate–vigorous intensity (SUCRA score, 83.75) was the best for diastolic blood pressure control.

#### 3.4.5 Network meta-analysis results for health-related quality of life

In the network meta-analysis of health-related quality of life, nineteen studies on the effect of exercise modalities and sixteen studies on different exercise intensities were included. The network plots of exercise modality (Figure 2G; Figure 2H) were presented for mental- and physical health-related quality of life.

We did not conduct a network analysis about the effect of different exercise intensities on health-related quality of life. All the included studies compared the effect of different exercise intensities with that of the control group, and no study compared the effect of various training intensities on health-related quality of life. Therefore, it was unable to form a connected network and cannot satisfy the connectivity assumption of network analysis (Higgins et al., 2012; Watt et al., 2019).

In the network meta-analysis of mental health-related quality of life, none of the exercise training modalities was more effective than those of the control group (Figure 3I). CT (MD = 4.46; 95% CI, −2.29–11.51), RT (MD = 5.62; 95% CI,−3.57–15.18), and AT (MD = 3.95; 95% CI, −1.49–10.00) were not effective compared with those in the control group (Figure 3I). EMS (MD = 6.32; 95% CI, −6.08–19.04) had no significant effect on mental health quality than in the control group. EMS had the highest probability (SUCRA score, 67.48) and was the best treatment for mental health-related quality of life.

In the network meta-analysis of physical health quality, all exercise modalities, except AT, were statistically equivalent to the control group (Figure 3J). CT (MD = 4.33; 95% CI, −4.21–13.02), RT (MD = 9.13; 95% CI, 1.12 to −19.59) had no statistical effect than the control group. AT (MD = 7.26; 95% CI, 0.33–13.8) was more effective than the control group (Figure 3J). EMS (MD = −4.95; 95% CI, −20.73 to 10.91) had no significant effect on physical health quality than the control group. RT had the probability (SUCRA score, 76.79) to be the best treatment.

#### 3.4.6 Publication bias and sensitivity analysis for each outcome

The comparison-adjusted funnel plots were symmetrically distributed and showed no publication bias. Egger^’^s test revealed no publication bias, and the *p-*value was higher than 0.05 for all outcomes, except for the exercise modality of 6MWT. There was evidence of publication bias (*p* = 0.02). The results are shown in Supplementary Appendix S10. We conducted a sensitivity analysis for all outcomes, and the results did not change when we excluded the high-risk studies. The result is presented in Supplementary Appendix S11.

## 4 Discussion

The network meta-analysis method has been used to explore the optimal exercise modality or intensity in some chronic diseases, such as obesity (Chang et al., 2021) and diabetes (Schwingshackl et al., 2014). Only a network meta-analysis (Scapini et al., 2019) has compared the effectiveness of different exercise modalities (AT, RT, and CT) with placebo on physical function, blood pressure, and hemodialysis efficiency among HD patients. That review needs to be updated, and it took no account of exercise intensity. Our research provided more comparisons and trials to comprehensively assess the effect of different exercise modalities. We also added a new outcome indicator, quality of life, in the current study. More importantly, our study is the first network meta-analysis to compare the effectiveness of different exercise intensities for HD patients.

In the current study, we compared the effect of various exercise modalities and intensities on different outcomes for hemodialysis patients. Most included trials last 4–6 months (3 sessions/weeks) and 30–50 min per session. The result showed some interesting findings, and we recommended that combined training (CT) and moderate–vigorous intensity are the optimal exercise modality and intensity for improving physical function and controlling blood pressure in HD patients.

Although the method of evaluating exercise intensity (light, moderate, and vigorous) has been widely used in the previous literature (Garber et al., 2011; Scharhag-Rosenberger et al., 2015), there are no standard definitions and descriptions of moderate–vigorous or light–moderate intensity exercise. However, physical activity of moderate–vigorous intensity is commonly recommended for health benefits in health guidelines (Tremblay et al., 2011). This ambiguity also makes it difficult for practitioners to offer accurate exercise advice. In order to improve the generalization of MV, a recent review by Brian (MacIntosh et al., 2021) suggested that the rating of perceived exertion might be a practical and effective measurement to define moderate–vigorous exercise. Therefore, the current study defined the moderate–vigorous intensity as RPE at 12–16 and the light–moderate intensity as RPE at 11–13. Meanwhile, based on the previous literature (Yoshioka et al., 2021a; Yoshioka et al., 2021b), which conducted exercises on chronic kidney diseases, we defined the MV intensity as higher than 3.0 metabolic equivalents.

Evidence has shown that moderate–vigorous intensity of exercise might benefit kidney function and physical function for patients with chronic diseases. A cross-sectional study (Hara et al., 2021) of 66,603 Japanese patients demonstrated that replacing 1 h of sedentary behavior with moderate–vigorous physical activity (≥3.0 metabolic equivalents) could reduce the incidence of chronic kidney diseases by 3–4%. Some studies (Yoshioka et al., 2021a; Yoshioka et al., 2021b) demonstrated that a slight increase (10 min/day) in the time of moderate–vigorous intensity exercise (≥3.0 metabolic equivalents) contributes to maintaining skeletal muscle strength and isometric knee extension strength in patients with chronic kidney disease, especially attenuating bone density decline. It is to be noted that the amount of moderate–vigorous intensity of exercise might exert different effects. A 10-year cohort (Wang et al., 2021) of 403,681 individuals suggested that participants who performed exercise at moderate–vigorous intensity and those with 50–75% vigorous intensity exercise had 17% lower all-cause mortality than those with no vigorous exercise. Future work should focus on the amount of moderate–vigorous intensity exercise and whether the proportion of moderate or vigorous intensity exerts different impacts on individuals.

Our results indicated that exercise could positively affect 6MWT regardless of modality and intensity. The most remarkable improvement was observed in exercise mortality with combined exercise and moderate–vigorous intensity. This finding was in agreement with that of another traditional meta-analysis that showed that exercise could increase at least 60 m for 6MWT (Ferrari et al., 2020). The 6MWT could reflect the ability to perform daily life activities and assess the global exercise responses. The previous meta-analysis suggested that 6MWT was sensitive as VO_2_ to evaluate exercise capacity and the effect of exercise among hemodialysis patients (Huang et al., 2019). Another meta-analysis (Gomes Neto et al., 2018) showed that all types of exercise improved the 6MWT distance among hemodialysis patients. It is critical to improving physical function for HD patients, characterized as 6MWT, which has been an independent mortality predictor of increased cardiovascular events (Sietsema et al., 2004). A 3-year follow-up showed that an increase of 20 walked meters in 6MWT among HD patients can reduce all-cause death by 12%, all-cause hospitalizations by 4%, and fatal and non-fatal cardiovascular events by 7% (Torino et al., 2014).

Our result showed that exercise had a limited effect on Kt/V. The result was consistent with that of previous systematic reviews (Salhab et al., 2019; Scapini et al., 2019). This might mean that exercises, regardless of modality or intensity, might not be able to improve hemodialysis efficiency or Kt/V was not the optimal measurement to evaluate the effect of exercise on hemodialysis efficiency. Kt/V represents the clearance of urea, a small solute distributed across plasma membranes (Vanholder et al., 2019). Some studies used the removal of serum potassium, creatinine, β2-microglobulin, or phosphate rather than urea to evaluate HD efficiency (Sampaio et al., 2012; Orcy et al., 2014). A meta-analysis (Ferreira et al., 2019) of three to five studies revealed that aerobic exercise could decrease creatinine levels for HD patients. Another study also found that exercise has no effect on Kt/V but had a significant decrease in serum phosphorus, creatinine, and potassium (Pellizzaro et al., 2013). It is to be noted that a recent review demonstrated that exercise had limited effects on improved small-molecule clearance for HD patients (Kirkman et al., 2019). Since Kt/V has been used in most previous studies, we still choose Kt/V as the measurement for hemodialysis efficiency. Future trials could assess the effect of exercise on the clearance of middle molecules and protein-bound uremic toxins.

The prevalence of hypertension among HD patients was up to 70–90% (Bikos et al., 2018), and uncontrolled hypertension was strongly associated with cardiovascular diseases (Loutradis et al., 2017). Our result found that exercise modalities did not affect blood pressure control, similar to a recent meta-analysis (Huang et al., 2019), which showed that the effect size of exercise on SBP and DBP was −0.18 (−0.42, 0.07) and −0.23 (−0.69, 0.24), respectively. In contrast with our results, another meta-analysis showed that CT could significantly reduce diastolic BP, and AT can improve systolic BP (Ferrari et al., 2020). However, their research showed moderate heterogeneity (44%). Our analysis has low heterogeneity and yields more reliable results.

It is to be noted that our results showed that moderate–vigorous intensity exercise could significantly reduce systolic blood pressure (8 mmHg) and diastolic blood pressure (4 mmHg). Consistent with our results, an 8-year cohort (Diaz et al., 2015) study consisting of 1,311 participants with moderate–vigorous physical activity (≥3.5 metabolic equivalents) had lower hypertension incidence for African–Americans (hazard ratio 0.76; 95% CI 0.58–0.99) compared to those who do not perform exercise. Another research showed that a 2 mmHg decrease in systolic blood pressure could reduce coronary heart disease and stroke in adults with hypertension (Heiwe and Jacobson, 2014). These results might be helpful in management of HD patients.

Our result found that exercise did not affect the mental or physical quality of life among HD patients. Contrary to our results, a systematic review (Huang et al., 2019) suggested that an 8-week exercise regimen could reverse the reduction of physical and mental health-related quality of life (SMD = 0.34, 95% CI: 0.09–0.59 and SMD = 0.27, 95% CI: 0.02–0.51, respectively) among HD patients. However, the meta-analysis only included seven trials (139 participants), and the limited sample size might exist bias. Nevertheless, a review conducted by Gomes Neto et al. (2018) demonstrated that AT could not improve the whole quality of life for HD patients. Previous studies (Sheng et al., 2014; Chung et al., 2017) also showed that AT and RT did not have a significant difference in the mental health-related quality of life. The baseline quality of life scores might cause the discrepancy. Painter et al. (2000)showed that HD patients with baseline physical quality of life (SF-36) scores less than 34 were associated with more remarkable improvement but not found in those with scores higher than 34. We should focus on HD patients with lower baseline quality of life scores who might more easily be affected by exercise.

## 5 Strengths and limitations

The strength of our network meta-analysis is that it provided evidence to guide the exercise of clinical practice for HD patients, which integrated direct and indirect comparisons to compare various modalities and intensities. A significant strength is that we comprehensively estimated the effectiveness of exercise intensities for HD patients. The validated measurement was used to classify the exercise intensities. Moreover, we provided the ranking of different exercise training modalities and intensities based on health outcomes for HD patients. Our evidence can help clinicians to choose the optimal exercise modality and intensity for HD patients. Furthermore, we found no heterogeneity and inconsistency in global and local analysis.

However, there were also some limitations. Most included studies were performed in a single medical center with limited number of patients. Future trials are required to be performed in multiple centers. The included trials did not compare the duration and session of exercise, contributing to difficulty in ascertaining the optimal exercise dose within the current literature. Since most of the included studies failed to report adverse events, this study does not pay attention to the adverse effects of exercise. A meta-analysis with limited trials showed that exercises would not increase the rate of adverse events for HD patients (Chung et al., 2017). Few trials have reported the adherence of patients after the intervention. We failed to explore exercise adherence after the intervention. Clinicians should help patients develop long-term exercise habits.

## 6 Conclusion

The present network analysis included four exercise modalities and six exercise intensities. In conclusion, combined training was the most effective intervention among current exercise interventions, and moderate–vigorous intensity was the most effective intensity in improving 6MWT and blood pressure control. In addition, exercise might not affect Kt/V and quality of life. Clinicians could give the optimal exercise prescription even for hemodialysis patients at home. Supervision was also needed to ensure the right exercise intensity. Future research can be conducted to estimate the clinical effect of exercise duration for HD patients and provide more evidence for clinical practice.

## Abbreviations

ACSM, American College of Sports Medicine; RCT, randomized clinical trial; 6MWT, 6-min walk test; AT, aerobic training; RT, resistance training; CT, combined training; EMS, electrostimulation; CN, control group; BP, blood pressure; L, light intensity; LM, light–moderate; M, moderate; MV, moderate–vigorous; V, vigorous; A, according to the patient’s needs; MH, mental health; PH, physical health; MD, mean difference.
