# Supplementary material for: The optimal exercise modality and intensity for hemodialysis patients incorporating Bayesian network meta-analysis and systematic review
Source: Front Physiol. 2022 Sep 19;13:945465. doi: 10.3389/fphys.2022.945465 (PMC9527310; doi:10.3389/fphys.2022.945465)
Supplement: Supplementary file 1 [file Table1.DOC]

**The optimal exercise modality and intensity for hemodialysis patients incorporating Bayesian network meta-analysis and systematic review**

Contents of supplementary appendix

[Appendix 1. Search Strategy 3](#__RefHeading___Toc106897132)

[Appendix 2 The detailed descriptions of exercise intensity 4](#__RefHeading___Toc106897133)

[Appendix 3 The characteristics of Included Studies 6](#__RefHeading___Toc106897134)

[Appendix 4 Risk of bias assessment 18](#__RefHeading___Toc106897135)

[Appendix 5 Results from pairwise meta-analysis for each outcome: numbers, estimates, and heterogeneity 20](#__RefHeading___Toc106897136)

[Appendix 6 network meta-analysis of relative effect League Table for each outcome 22](#__RefHeading___Toc106897137)

[Appendix 7 Assessment of Heterogeneity results for each outcome 26](#__RefHeading___Toc106897138)

[Appendix 8 Assessment of incoherence results for each outcome: global and local 27](#__RefHeading___Toc106897139)

[Appendix 9 Network analysis results for hemodialysis efficiency 30](#__RefHeading___Toc106897140)

[Appendix 10 Comparison-adjusted funnel plot for each outcome from the network meta-analysis 31](#__RefHeading___Toc106897141)

[Appendix 11 Network sensitivity analysis for each outcome-SURCA for each outcome 33](#__RefHeading___Toc106897142)

[Appendix 12 PRISMA NMA Checklist of Items to Include When Reporting A Systematic Review Involving a Network Meta-analysis 38](#__RefHeading___Toc106897143)

**List of Abbreviations:**

ACSM: American College of Sports Medicine

RCT: randomized clinical trial

6MWT: 6 - minute walk Test

AT: aerobic training

RT: resistance training

CT: combined training

EMS: electro-stimulation

CN: control group

BP: blood pressure

L: light intensity

LM: light-moderate

M. moderate

MV: moderate-vigorous

V: vigorous

A: according to patient needs

MH: mental-related health

PH: physical-related health

# Appendix 1. Search Strategy

**PubMed**

#1 “Exercise” [Mesh] OR “Exercises” OR “Exercise, Physical” OR “Exercises, Physical” OR “Physical Exercise” OR “Physical Exercises” OR “Warm-Up Exercise” OR “Exercise, Warm-Up” OR “Exercises, Warm-Up” OR “Warm-up Exercise” OR “Warm-Up Exercises” OR “Exercise, Aerobic” OR “Aerobic Exercises” OR “Exercises, Aerobic” OR “Aerobic Exercise” OR “Exercise Therapy” [Mesh] OR “Therapy, Exercise” OR “Exercise Therapies” OR “Therapies, Exercise” OR “Resistance Training” [Mesh] exercise[MeSH] OR exercises OR resistance exercise OR resistance training[MeSH] OR strength training OR aerobic exercise OR aerobic exercises OR exercise aerobic OR aerobic training OR aerobic versus strength OR strength versus aerobic OR physical fitness[MeSH] OR physical training OR exercise therapy[MeSH]OR “Training, Resistance” OR “Strength Training” OR “Weight-Lifting Exercise Program” OR “Weight Lifting Exercise Program” OR “Weight-Bearing Strengthening Program” OR “Strengthening Programs, Weight-Bearing” OR “Weight Bearing Strengthening Program” OR “Weight-Bearing Strengthening Programs” OR “IMT” OR “EMS” OR “yoga” OR “combined training” OR “aerobic plus strength training” OR “aerobic plus resistance exercise” OR “aerobic plus resistance training” OR “concurrent training” OR “concurrent exercise” OR “electro-stimulation” OR “electro-stimulation”

#2 “End-Stage Kidney Disease” OR “Disease, End-Stage Kidney” OR “End Stage Kidney Disease” OR “Kidney Disease, End-Stage” OR “Chronic Kidney Failure” OR “End-Stage Renal Disease” OR “Disease, End-Stage Renal” OR “End Stage Renal Disease” OR “Renal Disease, End-Stage” OR “Renal Disease, End Stage” OR “Renal Failure, End-Stage” OR “End-Stage Renal Failure” OR “Renal Failure, End Stage” OR “Renal Failure, Chronic” OR “Chronic Renal Failure” OR “ESRD” OR “Renal Dialysis” [Mesh] OR “Renal Dialyses” OR “Dialysis, Renal” OR “Hemodialysis” OR “Hemodialyses”

#3 randomized controlled trial[pt] randomized controlled trials[mh] OR random allocation[mh] OR double-blind method[mh] OR single-blind method[mh] OR clinical trial[pt] OR clinical trials[mh] OR ("clinical trial"[tw]) OR ((singl*[tw] OR doubl*[tw] OR trebl*[tw] OR tripl*[tw]) AND (mask*[tw] OR blind*[tw])) OR ("latin square"[tw]) OR placebos[mh] OR placebo*[tw] OR random*[tw] OR research design[mh:noexp] OR follow-up studies[mh] OR prospective studies[mh] OR cross-over studies[mh] OR control*[tw] OR prospectiv*[tw] OR volunteer*[tw] NOT (animal[mh] NOT human[mh])

# 1 AND #2 AND #3

# Appendix 2 The detailed descriptions of exercise intensity

Light, Moderate and Vigorous were defined according to ACSM Recommendations^1,2^. The indicators of Exercise intensity are HR (heart rate), HRR (heart rate reserve), VO2max, or some subjective parameters such as the rating of perceived exertion (RPE). The resistance exercise was classified by repetition maximum (RM). The moderate-vigorous intensity was defined as RPE at 12-16, and the light-moderate intensity was defined as RPE at 11-13 in our study. Meanwhile, based on the previous literatures^3,4^, which conducted exercises on chronic kidney diseases, then we also defined the Moderate-vigorous intensity as higher than 3.0 metabolic equivalents. We added an intensity that was according to the patient’s need. Some exercise program was designed so that participants could exercise at an intensity determined by their perceived exertion level. There was no specific exercise velocity or target heart rate for patients to achieve^5,6^.

eTable1: The descriptions of exercise intensity

| Cardiorespiratory endurance exercise | | | | | |  | Resistance exercise |
| --- | --- | --- | --- | --- | --- | --- | --- |
| Intensity | VO_2max_  (%) | HR _max_  (%) | HRR  (%) | RPE: Borg scale (rating on 6-20) | RPE: Borg scale (rating on 1-10) | METs | RM(repetition maximum) |
| Light | ＜46 | ＜65 | ＜40 | ≤11 | ＜3 | ＜2.9 | 20%-50% |
| Moderate | 46-64 | 65-76 | 40-60 | 12-13 | 3-8 | 3-5.9 | 50%-75% |
| Vigorous | ＞64 | ＞76 | ＞60 | ≥14 | ≥9 | 6-8.7 | ≥75% |

* VO_2max_, maximal oxygen uptake; HRmax, maximal Heart rate; HRR, Heart reserve; RPE, ratings of perceived exertion. METS, metabolic equivalents.

# Appendix 3 The characteristics of Included Studies

eTable2: The characteristics of Included Studies

| **Study characteristic** | | | | | **Intervention** | | | | | |
| --- | --- | --- | --- | --- | --- | --- | --- | --- | --- | --- |
| **Author,**  **year** | **Country/Region** | **Sample size** | **Age (years)**  **Mean±S** | **Hemodialysis time (months) Mean±SD** | **Exercise**  **modality** | **Intervention details** | **Exercise intensity** | **Exercise frequency (times/week)** | **Duration/session (min)** | **Duration (week)** |
|  |  | **intervention/control group** | | |  |  |  |  |  |  |
| Painter et al. 2002^7^ | USA | 10/14 | 47.6±11.9/43.3±9.8 | 23.1±24.6/61.8±72.9 | AT | 10-15min non-resistance exercise, 30min ergometer Cycling. The intensity was prescribed using the RPE rating (12-14) with a regular heart rate to assure at least 70% HR max. | Moderate to vigorous | 3/week | 50 | 20 |
| De Paul et al. 2002^8^ | Canada | 20/18 | 55±16/54±14 | 4.2±4.8/4.6±4.5 (year) | CT | 20min ergometer, cycling at 13 to 14 RPE, at around 50 rpm, 3 sets of 10 reps of hamstring and quadriceps exercises on weight machines, start at 50% of 5RM, the percentage increased to a maximum at 125% of 5 RM. | Moderate to vigorous | 3/week | 30 | 12 |
| Tsuyuki et al 2003^9^ | Japan | 17/12 | 40.1±11.9/39.7±10.7 | 2.1±2.5/2.7±2.6 (year) | AT | 30min ergometer cycling, jogging, and walking, intensity maintain at 50 to 60% HR max | Light | 3/week | 30 | 20 |
| Molsted et al. 2004^10^ | Denmark | 22/11 | 59(25-58)/48(23-58) | 23.5(4-720)/17(3-60) | CT | 10min warm-up, 20 to 30min of strength exercises and aerobic exercises (such as step and circuit training), 15 to 20 min ergometer cycling; intensity maintain at 14 to 17 RPE. | Vigorous | 2/week | 50 | 20 |
| Van Vilsteren et al. 2005^11^ | Netherlands | 53/43 | 52(15)/58(16) | 1.19±0.22/1.23±0.22(year) | CT | 5 to 10min warm-up, 20min of calisthenics exercise, step aerobics, flexibility, low-intensity resistance exercises, 5 to 10 min cooldown, 20 to 30 min of cycling on pedals coupled to the hemodialysis chair, intensity maintained at 12 to 16 RPE. | Moderate to vigorous | 2-3/week | 20-30 | 12 |
| Cheema et al. 2007^12^ | Australia | 24/25 | 62.6±14.2 | 2.2(0.3-16.7)(year) | RT | 2 sets of 8 reps; five upper limb exercises using free-weight dumbbells, five lower limb exercises using ankle weights or elastic resistance bands, and one abdominal exercise, upper body exercises that were performed using free-weight dumbbells; intensity maintained at 15 to 17 RPE | Vigorous | 3/week | NR | 12 |
| Kopple et al. 2007^13^ | USA | 20/20/20/20 | 45.9±4.1/46±2.7/42.7±3.8/41.3±3.3 | 45.9±14.1/51.9±12.4/38.3±5.8/51.4±21 | AT/RT/CT | AT: 5-10min warm-up, 40min ergometer cycling, the intensity is at 50% of their peak oxygen consumption; RT: apparatus for combined leg extension and flexion, and leg press and plantar flexion; 3 sets of 6 to 8 reps and resistance to 70% to 80% of 5RM; CT: performed a combination of approximately one half of the AT and RT work effort. | AT: Moderate  RT： Moderate  CT: Moderate | 3/week | AT:50 | AT:20.7  RT:21.2  CT:21.5 |
| Toussaint et al. 2008^14^ | Australia | 9/10 | 67(60-83)/70(28-77) | 35±31/72±56 | AT | 30 min bicycle ergometers, the intensity determined by their perceived exertion level. | According to patient’s needs | 3/week | 30 | 12 |
| Sakkas et al. 2008^15^ | Greece | 7/7 | 59±16 | 2.2±1.2/2.1±0.7(year) | AT | 45min continuous cycling using a bedside cycle ergometer, 5min warm-up, 5min cool down, the intensity was set between 65%-75% of their maximum power capacity (watts). | Moderate | 3/week | 45 | 16 |
| Ouzouni et al. 2009^16^ | Greece | 20/15 | 47.4(15.7)/50.5(11.7) | 7.7(7.0)/8.6(6.0) (year) | CT | 30min ergometer cycling (5min warm-up, 20min cycling, 5min cooldown, 30 min of strengthening and flexibility exercises with elastic bands and limb weights; intensity maintain at 13 to 14 RPE. | Moderate to vigorous | 3/week | 60-  90 | 40 |
| Afshar et al. 2010^17^ | Iran | 7/7/7 | 50.7±21.06/51±16.4/53±19.4 | 25.71±7.61/24.86±18.69/24.86±15.44 | AT/RT | AT: 10 to 30 min ergometer cycling at 12 to 16 RPE;  RT: 3 sets of 8 reps at 60% of 3RM of knee extension-flexion and hip abduction flexion at 15 to 17 RPE with ankle weights. | AT:  Moderate to vigorous  RT:  Vigorous | 3/week | AT:10-30min  NR | 8 |
| Reboredo et al. 2010^18^ | Brazil | 11/11 | 49.6±10.6/43.5±12.8 | 41.9±42.4/60.1±54.4 | AT | 5min Warm-up, 35min conditioning and cool down, electromagnetic cycle ergometer, the load was set between 4-6 Borg-modified scale. | Moderate | 3/week | 35 | 12 |
| Koh, K P et al. 2010^19^ | Australia | 15/15/16 | 52.3±10.9/52.1±13.6/51.3±14.4 | NR | AT | 45min ergometer cycling at 12 to 13 RPE. | Moderate | 3/week | 30-45 | 24 |
| Wilund et al2010^20^ | USA | 8/9 | 60.8±3.2/59.0±4.9 | 44.6±12.2/63.3±8.7 | AT | 45 min ergometer cycling at 12 to 14 RPE. | Moderate to vigorous | 3/week | 45 | 16 |
| Dobsak et al 2012^21^ | Czech  Republic | 11/11/10 | 58.2±7.2/64.5±8.1/60.1±8.2 | 4.1±2.1/3.9±1.8(year) | AT/EMS | AT: 2 sets of 20min ergometer cycling at 60% of individual Wpeak determined by the ergometric test, 5 min cool down.  EMS: stimulation (10 Hz) of leg extensors was applied for 60 min | Moderate | 3/week | AT:40min EMS:60 | 20 |
| Giannaki et al. 2013^22^ | Greece | 12/12 | 59.2±11.8/58.0±10.7 | 24.0±15/30±26 | AT | 45min exercise, Cycling in a recumbent cycle ergometer at an intensity of 60%-65% of the patient maximal exercise capacity (in Watts). | Moderate | 3/week | 45 | 24 |
| Mohseni et al. 2013^23^ | Iran | 25/25 | 53±14/56±11 | 26±11/24±14 | AT | 15 min aerobic movement exercise of a range of motion (rotating wrist 40rpm, 20 times flexion and extension of the ankles. | None | 2-3/week | NR | 8 |
| Pellizzaro et al. 2013^24^ | Brazil | 11/14/14 | 43±13.8/48.9±10.1/51.9±11.6 | 60(24-132)/54(10.7-120)/54(12-78) | RT | 50% of 1RM, 3 sets of 15 knee extension repetitions, resting for 60 s. | Moderate | 3/week | NR | 10 |
| Kirkman et al. 2014^25^ | UK | 9/10 | 48±18/58±15 | 46±54/66±47 | RT | 80% 1 RM, 3 sets 8-10 reps. | Vigorous | 3/week | NR | 12 |
| Wu et al. 2014^26^ | China | 32/33 | 45(37-48)/44(41-50) | 55.5±37.3/39.8±29.7 | AT | Intensity maintains at 12-16RPE; 5 min warm-up, 10-15min exercise. | Moderate to vigorous | 3/week | 15-20 | 12 |
| Groussard et al. 2015^27^ | France | 10/10 | 66.5±4.6/68.4±3.7 | 41.2±8.1/36.6±8.2 | AT | 30min ergometer cycling (5min warm-up,5 min cooldown), and the workload was set at 55% to 60% of the peak power output | NR | 3/week | 30 | 12 |
| Roxo et al  2016^28^ | Brazil | 20/20 | 46.40±15.43/  54.65±19.93 | 68.80±46.90  /46.15±41.40 | EMS | Tolerance of each patient, 24 sessions, 50Hz frequency for 2 s, rest for 10 s. | NR | 3/week | 30 | 8 |
| Liao et al. 2016^29^ | Taiwan | 20/20 | 62±8/62±9 | 71±46/83±71 | AT | 30min, 5min warm-up, 20min ergometer cycling at 12 to 15RPE, 5 min cool down. | Moderate to vigorous | 3/week | 30 | 12 |
| Thompson et al. 2016^30^ | Canada | 8/7/8/8 | 66.9(55.8-82.4)/59.7(45.9-81.4)/60.3(54.7=68.4) | 3.7(2.4-4.6) /2.8(2.0-4.0)/2.9(0.7-2.3)/3.3(1.2-6.2) (year) | AT/RT/CT | AT: 5 min warm-up. 15min ergometer cycling at 12 to 14 RPE increased by 2.5min/week,5 min cooldown,  RT: 3 sets of 10 to 15 reps, knee extension, knee flexion, and hip flexion and hip abduction at 12 to 14RPE;  CT: Exp1 followed by Exp2 at each session/ | AT: Moderate to vigorous; RT: Moderate to vigorous  CT: Moderate to vigorous | 3/week | 30 | 12 |
| Pomidori et al. 2016^6^ | Italy | 22/20 | 63±15/69±10 | 7±8/5±6years | AT | Two daily 10-min walking sessions: a prescribed walking speed to be maintained at home with a metronome. The intensity was set below the self-selected Speed. | According to the warm-up patient’s needs | 3/week | 10 | 24 |
| Frih et al. 2017^31^ | Tunisia | 28/22 | 64.2±3.4/65.2±3.1 | 72.7±12.7/73.6±13.4 | CT | 60min: 10min warm-up (isolated mobilizing to gross mobilizing exercises) dynamic closed- and open-chain strengthening exercises (quadriceps, pectoral, triceps, biceps, and hamstrings, start at 50% of their 1RM and 12 to 15 repetitions for each exercise, the load increased by 5% of 1RM monthly), ergometer cycling and treadmill walking for 20min,10min cool down/no exercise | Moderate | 4/week | 60 | 16 |
| Schardong et al 2017^32^ | Brazil | 11/10 | 59(45-72)/64.5(57.5-67.75) | 47.10±44.61/56.18±61.60 | EMS | EMS: Tolerance of each patient, Isometric exercise, 80Hz frequency for 10 s, Rest starting with 50 s and reducing by 10 s every 2 wk. | None | 3/week | 20-30 | 8 |
| Abreu et al. 2017^33^ | Brazil | 25/19 | 45.7±15.2/42.5±13.5 | 71.2±45.5/70.1±49.9 | RT | 3 sets of 10 repetitions. Four different movements in lower limb exercises with ankle cuffs and elastic band resistance; The intensity was based on an adaptation of the 1 RM test. With the initial intensity set at 60% of 1 RM. | Moderate | 3/week | 30 | 12 |
| Cooke et al. 2018^34^ | Canada | 10/10 | 58.2±17.2/52.5±15.4y | NR | AT | Pedaling exercise, 12-16RPE. | Moderate to vigorous | 3/week | 30-60 | 12 |
| Rosa et al. 2018^35^ | UK | 28/24 | 55.7±14.03 | 1.54±1.26/2.35±1.66(year) | RT | 11 types of exercise (stand row, biceps curls, dumbbell, hip flexion, etc.). 2 sets of 15-20 reps in an RM | Vigorous | 3/week | 40-50 | 12 |
| McGregor et al. 2018^36^ | UK | 17/16/18 | 51.5(42.3,60.6)/52.1(44.2,59.9)/54.3(46,62.5) | 56.4(36.1,76.6)/48.1(26.2,70)/49.3(29.6,69) | EMS/AT | EMS: Low-frequency electrical muscle stimulation, at maximum tolerable intensity  AT: 12-14RPE, 5 min warm-up, 40-50 min exercise, 5 min cool down. | Moderate to vigorous | 3/week | 50-60 | 10 |
| Tadashi Suzuki et al. 2018^37^ | Japan | 13/13 | 66.2±12.8/65.1±8.1 | 28.1±24.2/30.4±23.6 | EMS | The training was performed using a handheld muscle stimulator, silicon-rubber electrode bands, and 5.5 cm in width, were wrapped around the waist and the bilateral distal femurs and ankles to stimulate the gluteal and upper- and lower-leg muscle groups. The stimulator was designed at a frequency of 20HZ with a pulse width of 250μs. Each duty cycle included a 5-s stimulation period. | None | 3/week | 120 | 8 |
| Dong et al. 2019^38^ | China | 21/20 | 59.0(32.5,66.5)/62.5(50.5,70.0) | 69.0(31.5,87.5)/57.5(32.5,86.5) | RT | Tolerance of each patient, 5 min warm-up  ankle weight (0 kg), Quadriceps training (low intensity), ankle weight (+0.5 kg) per week, 5kg  Pressure on the elastic ball and maximally (3-5 s) - 10 × 10 cycles repeatedly. | RT: According to patients’ needs | 3/week | NR | 12 |
| Fernandes et al. 2019^39^ | Brazil | 20/19 | 44.25±11.30/ 42.63±11.16 | 6.65±4.70/7.16±3.78(year) | AT | 50-70% HR max, 10 min warm-up (active upper and lower limb exercises), 30 min exercise, 10 min cool down. | Light to moderate | 3/week | 50 | 8 |
| Martins do Valle et al 2019^40^ | Brazil | 12/12 | 49.3±12.4/60.4±10.6 | 6.8±11.4/3.9±12.5(year) | RT | Borg rating scores between 3-5, Lower limb stretching exercises; Strengthening exercises (upper and lower limbs). | Light | 3/week | NR | 12s |
| Hatef et al. 2020^41^ | 2020 | 28/27 | 52.07± 11.30 /53.96 ±10.01 | 3.61 ±2.69 /4.52± 4.71(year) | CT | Warm-up, 20min walk, and cool down, 11-13RPE. | Light to moderate | 3/week | 30 | 8 |
| Huang et al. 2020^42^ | China | 16/16 | 43.81±10.25/37.63±10.31 | 26±29.75/43±89 | CT | 5min warm-up, cool down, and 30min cycling at an RPE of 12-14 | Moderate to vigorous | 3/week | 80 | 24 |
| Mei-Ling Yeh et al 2020^43^ | Taiwan | 30/32 | 57.87±13.21/53.91±12.60 | 63.47±71.98/78.28±63.95 | CT | Warm-up, mix exercise, cool-down exercise, cycling ergometer, resistance modalities, and exercise intensity was measured using the perceived exertion scale. The intensity was maintained at the perceived exertion scale of 12-14. | Moderate to vigorous | 3/week | 30 | 12 |
| Assawasaksakul et al. 2020^44^ | USA | 6/6 | 52.5±12.9/53.7±17.2 | 105(30,155.3)/66.5(20.0,89.8) | CT | 5min stretching, warm-up by cycling at 40 pedal revolutions per minute, the intensity was over 60%-70% of maximal heart rate. | Light to moderate | 3/week | 60 | 24 |
| Myers et al  2021^45^ | USA | 13/15 | 66.3±7.6/66.2±6.7 | 4.25±3.9/4.05±3.9(year) | CT | Participants were given handheld weights and Thera-bands in accordance with their capabilities and portable cycle ergometers for home use, a combination of the control group. Aerobic exercise and resistance exercise. Daily activity logs were used to record. The intensity was set at 70%-80% of HR reserve and 12-14 RPE. | Vigorous | 7/week | 45 | 12 |
| Matthew et al. 2021^46^ | UK | 50/51 | 55.5±15.5/58.9±14.9 | 1.2(0.5-3.7)/1.3(0.4-3.2)(year) | RT | Participants in the exercise group undertook cycling on specially adapted and calibrated cycle ergometers 3 times a week during dialysis, aiming for 30 minutes  continuous cycling at a rating of perceived exertion (RPE) of 12 to 14, complete 30 minutes of continuous cycling until this target was achieved | Moderate to vigorous | 3/week | 30 | 24 |
| Gadelha et al. 2021^47^ | Brazil | 22/20 | 68.1±3.3/65.6±2.9 | 58.4±9.7/54.3±11.5 | RT | It involved the following exercises: chest press, squat, unilateral row, unilateral knee extension, unilateral knee flexion, unilateral shoulder press, hip thrust, biceps curl, unilateral hip adduction, unilateral hip abduction, elbow extension with dumbbells, and seated calf raise. Training intensity was at an RPE scale of 5–6 for the first 12 weeks and 7–8 over the final 12 weeks | Moderate | 3/week | 40 | 24 |
| Chia-Huei et al 2021^48^ | Switzerland | 32/32 | 62.0±9.5./62.1±12.3 | 6.7±5.7/6.2±5.1 | AT | The intensity of exercise was set at a rating of RPE score (12–14); the duration of each exercise session consisted of a 5-min warm-up, 20-min endurance, and 5-min cool-down phase. The exercise occurs at least one to two hours after a meal. | Moderate to vigorous | 3/week | 30 | 12 |
| Borja et al. 2021^49^ | Spain | 36/34 | 67.2±13.3/67.2±15.9 | None | AT | 5-minute warm-up, with a 30-minute aerobic training with the use of a cycle ergometer, When finished, par­ticipants were instructed to “cool down” with active joint mobilizations and stretching exercises. Exercise intensity was set as an RPE score of 12. | Moderate | 3/week | 60 | 16 |
| Ekaterini et al. 2022^50^ | Greece | 12/12 | 58.0±14.3/57.4±14.3 | 8.2±4.0/8.5±5.0 | AT | The program included a 5-min warm-up (lower limb stretching), 30–50-min cycling, and a 5-min recovery (stretching of lower limbs). The duration of cycling gradually increased over time according to each patient’s ability and reached an hour of active cycling. The exercise intensity was at a rating of RPE score between 13 and 14. | Moderate to vigorous | 3/week | 60 | 24 |
| Sunki et al. 2022^51^ | Republic of Korea | 18/21 | 57.61±13.69/56.76±12.32 | 2.38±2.72/5.74±3.99y | AT | stretching warm-up phase (5 minutes), the main exercise phase (30–60 minutes), and a cool-down phase (5 minutes). During the main phase, exercise cycle ergometer exercise was safely performed during the first 1 to 1.5 hours of each di­alysis session without cardiac decompensation. Exercise intensity was at a rating RPE score between 12–15. | Moderate to vigorous | 3/week | 40-70 | 12 |

Note: AT, aerobic training; RT, resistance training; CT, combined therapy; EMS electro-stimulation, CN, control group; RPE: the rating of perceived exertion; HR: heart rate; RM: repetition maximum.

# Appendix 4 Risk of bias assessment


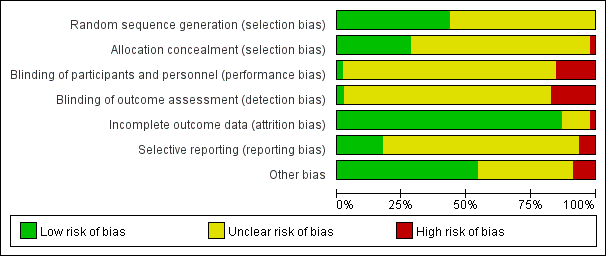


e-Figure 1. Risk of bias graph of included studies


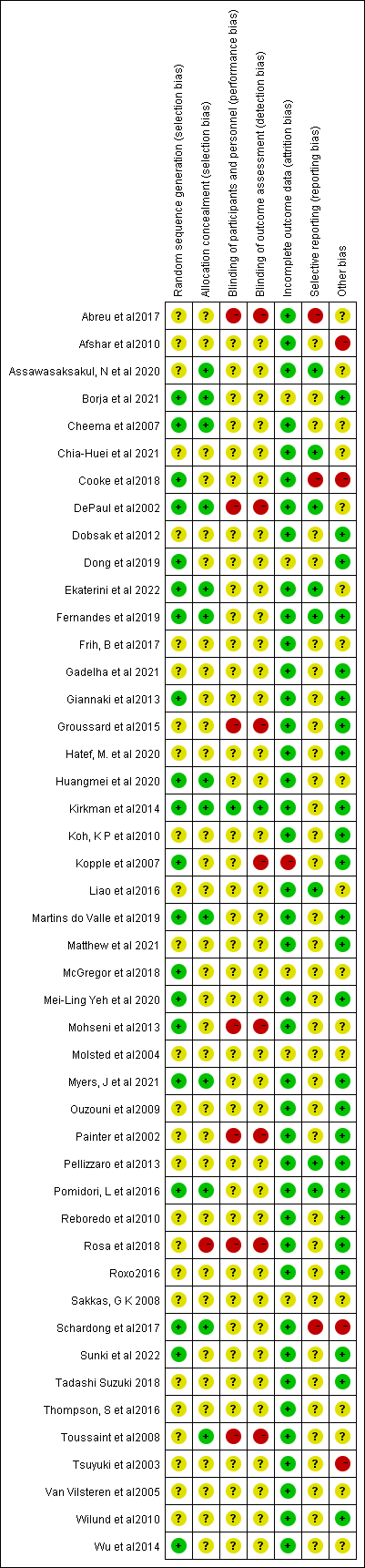


e-Figure 2. Risk of bias summary for included studies

# Appendix 5 Results from pairwise meta-analysis for each outcome: numbers, estimates, and heterogeneity

eTable3: Results from pairwise meta-analysis for each outcome

| Comparison | No. of studies | MD(95%CI) | I^2^ (%) |
| --- | --- | --- | --- |
| 6MWT -Exercise modality | | | |
| AT vs. CN | 7 | 3.3(0.7,6.0) | 0 |
| CT vs. CN | 6 | 4.9(3.1,6.7) | 0 |
| EMS vs. CN | 2 | 2.3 (-2.1,6.6) | 0 |
| RT vs. CN | 6 | 2.5(0.56,4.5) | 0 |
| RT vs. CT | 2 | -0.19(-15.0,15.0) | 0 |
| 6MWT -Exercise intensity |  |  |  |
| M vs. CN | 4 | 5.0(1.8,7.7) | 0 |
| MV vs. CN | 4 | 4.5(1.9,7.1) | 4.8 |
| MV vs. L | 3 | 4.6(0.80,8.4) | 0 |
| V vs. CN | 4 | 1.6(-1.3,4.0) | 0 |
| Kt/V -Exercise modality | | | |
| AT vs. CN | 12 | -0.07(-0.19,0.05) | 84.5 |
| RT vs. AT | 2 | -0.07 (-0.25,0.10) | 75.1 |
| CT vs. CN | 3 | 0(-0.20,0.22) | 88.7 |
| EMS vs. CN | 2 | 0.04(-0.22,0.32) | 88.0 |
| RT vs. CN | 7 | 0(-0.15,0.14) | 92.0 |
| Kt/V-Exercise intensity |  |  |  |
| M vs. CN | 12 | 0(-0.09,0.11) | 91.0 |
| MV vs. CN | 5 | 0.10(-0.07,0.25) | 10.4 |
| V vs. CN | 2 | 0(-0.24,0.29) | 0 |
| SBP-Exercise modality | | | |
| AT vs. CN | 12 | -6.1(-13.0,0.52) | 65.8 |
| CT vs. CN | 6 | -8.0(-17.0,1.3) | 75.7 |
| EMS vs. CN | 3 | 0.89(-10.0,13.0) | 90.0 |
| RT vs. CT | 2 | -1.2(-21.0,18.0) | 0 |
| RT vs. CN | 2 | 1.2(-17.0,18.0) | 0 |
| SBP-Exercise intensity |  |  |  |
| CN vs. L | 2 | -2.1(-22.0,17.0) | 0 |
| MV vs. CN | 13 | -7.9(-15.0,-1.7) | 68.7 |
| DBP-Exercise modality | | | |
| CN vs. AT | 12 | 2.7(-1.2,6.7) | 63.9 |
| CT vs. CN | 5 | -2.9(-8.7,3.4) | 73.9 |
| EMS vs. CN | 3 | 1.8 (-5.1,9.1) | 21.0 |
| RT vs. CT | 2 | -0.24(-12.0,11.0) | 0 |
| RT vs. CN | 2 | -0.91(-11.0,9.5) | 0 |
| DBP-Exercise intensity |  |  |  |
| MV vs. CN | 13 | -4.4(-8.4,-0.86) | 69.8 |
| MH-Exercise modality | | | |
| CN vs. AT | 8 | -3.8(-10.0,2.2) | 60.0 |
| CT vs. CN | 4 | 3.6(-4.1,11.0) | 58.8 |
| RT vs. CN | 3 | 5.7(-5.4,17.0) | 70.3 |
| MH-Exercise intensity |  |  |  |
| M vs. CN | 3 | 3.6(-7.6,16.0) | 84.0 |
| MV vs. CN | 7 | 2.4(-4.6,9.4) | 66.6 |
| PH-Exercise modality | | | |
| CT vs. CN | 4 | 3.6(-5.9,13.0) | 0 |
| RT vs. CN | 3 | 8.7(-3.0,21.0) | 86.7 |
| AT vs. CN | 8 | 6.9(-23.0,11.0) | 77.4 |
| PH-Exercise intensity |  |  |  |
| M vs. CN | 3 | -0.52(-12.0,11.0) | 0 |
| MV vs. CN | 7 | 5.8(-1.2,13.0) | 82.9 |

# Appendix 6 network meta-analysis of relative effect League Table for each outcome

6MWT- exercise modality

| AT |  |  |  |  |
| --- | --- | --- | --- | --- |
| **3.53(1.09,6.01)** | CN |  |  |  |
| -1.34(-4.26,1.66) | **-4.89(-6.60,-3.02)** | CT |  |  |
| 1.33(-3.72,6.24) | -2.23(-6.61,2.15) | 2.60(-2.14,7.31) | EMS |  |
| 1.04(-2.07,4.12) | **-2.50(-4.48,-0.61)** | 2.39(-0.34,4.92) | -0.28(-5.14,4.68) | RT |

6MWT- exercise intensity

| A |  |  |  |  |  |  |
| --- | --- | --- | --- | --- | --- | --- |
| 3.62(-3.24,10.45) | CN |  |  |  |  |  |
| 1.11(-6.38,8.75) | **-2.55 (-5.39,0.52)** | L |  |  |  |  |
| -0.67(-9.04,7.50) | -4.30(-9.01,0.53) | -1.77(-7.35,3.79) | LM |  |  |  |
| -1.09(-8.38,6.38) | **-4.71(-7.34,-1.70)** | -2.17 (-5.86,1.74) | -0.42(-5.86,5.31) | M |  |  |
| -1.75(-9.03,5.49) | **-5.36(-7.73,-2.99)** | **-2.82(-5.80,-0.02)** | -1.08 (-6.35,4.20) | -0.66(-4.48,2.75) | MV |  |
| 2.12(-5.14,9.42) | -1.53(-4.04,1.24) | 1.00(-2.91,4.97) | 2.78(-2.55,8.25) | 3.23(-0.79,6.83) | **3.82(0.39,7.44)** | V |

Kt/V- exercise modality

| AT |  |  |  |  |
| --- | --- | --- | --- | --- |
| 0.07(-0.05,0.19) | CN |  |  |  |
| 0.06(-0.17,0.30) | -0.01(-0.22,0.20) | CT |  |  |
| 0.03(-0.26,0.31) | -0.04(-0.32,0.22) | -0.04(-0.38,0.30) | EMS |  |
| 0.07(-0.10,0.25) | 0.00(-0.14,0.15) | 0.01(-0.23,0.26) | 0.05(-0.25,0.36) | RT |

Kt/V –exercise intensity

| A |  |  |  |  |  |  |
| --- | --- | --- | --- | --- | --- | --- |
| 0.16(-0.19,0.51) | CN |  |  |  |  |  |
| -0.09(-0.59,0.42) | -0.25(-0.61,0.11) | L |  |  |  |  |
| 0.26(-0.23,0.75) | 0.10(-0.24,0.44) | 0.35(-0.15,0.85) | LM |  |  |  |
| 0.15(-0.22,0.52) | 0(-0.11,0.09) | 0.24(-0.13,0.61) | -0.11(-0.46,0.25) | M |  |  |
| 0.07(-0.31,0.46) | -0.09(-0.24,0.06) | 0.16(-0.23,0.55) | -0.19(-0.56,0.19) | -0.08(-0.26,0.11) | MV |  |
| 0.13(-0.31,0.58) | -0.03(-0.29,0.24) | 0.22(-0.22,0.66) | -0.12(-0.56,0.30) | -0.02(-0.29,0.26) | 0.06(-0.23,0.35) | V |

Systolic blood pressure- exercise modality

| AT |  |  |  |  |
| --- | --- | --- | --- | --- |
| -6.21(-12.48,0.04) | CN |  |  |  |
| 0.83(-9.54,10.65) | 7.02(-1.38,15.00) | CT |  |  |
| -5.75(-17.78,5.46) | 0.44(-10.59,10.82) | -6.60 (-19.91,6.66) | EMS |  |
| -3.08(-16.77,10.74) | 3.13(-9.50,15.96) | -3.95(-16.88,9.70) | 2.64(-13.43,19.89) | RT |

Systolic blood pressure- exercise intensity

| A |  |  |  |  |  |  |
| --- | --- | --- | --- | --- | --- | --- |
| -0.52(-25.39,24.44) | CN |  |  |  |  |  |
| 1.53(-28.41,31.75) | 2.04(-14.71,18.75) | L |  |  |  |  |
| 0.54(-32.29,33.33) | 1.12(-20.19,22.06) | -0.95(-28.06,25.88) | LM |  |  |  |
| -0.39(-35.69,34.71) | 0.13(-24.63,25.03) | -1.91(-31.98,27.94) | -0.97(-33.51,31.61) | M |  |  |
| 6.83(-18.46,32.96) | **7.33(1.25,14.08)** | 5.28(-10.92,22.34) | 6.19(-15.23,28.77) | 7.22(-18.16,33.08) | MV |  |
| -1.24(-36.83,34.29) | -0.78(-26.0,24.61) | -2.76(-33.03,27.72) | -1.83(-34.67,31.13) | -0.81(-36.40,34.54) | -8.11(-34.70,17.81) | V |

Diastolic blood pressure- exercise modality

| AT |  |  |  |  |
| --- | --- | --- | --- | --- |
| -2.39(-6.22,1.28) | CN |  |  |  |
| 0.36(-6.40,6.58) | 2.75(-2.87,8.03) | CT |  |  |
| -3.06(-10.45,3.70) | -0.67(-7.44,5.59) | -3.44(-11.94,4.96) | EMS |  |
| -0.30(-8.71,8.04) | 2.05(-5.56,9.86) | -0.67(-8.45,7.55) | 2.74(-7.02,13.14) | RT |

Diastolic blood pressure- exercise intensity

| A |  |  |  |  |  |  |
| --- | --- | --- | --- | --- | --- | --- |
| 4.98(-5.21,15.22) | CN |  |  |  |  |  |
| 3.96(-10.36,18.09) | -1.00(-11.04,8.77) | L |  |  |  |  |
| 4.38(-11.91,20.62) | -0.59(-13.22,12.00) | 0.42(-15.60,16.67) | LM |  |  |  |
| 6.49(-6.59,19.53) | 1.46(-11.09,14.14) | 2.48(-13.36,18.74) | 2.10(-15.80,20.00) | M |  |  |
| 9.13(-1.60,20.08) | **4.13(0.64,7.93)** | 5.14(-4.47,15.30) | 4.73(-8.21,18.03) | 2.66(-10.34,15.90) | MV |  |
| 3.00(-16.27,22.14) | -1.96(-18.16,14.20) | -0.99(-19.83,18.06) | -1.36(-22.03,19.01) | -3.46(-24.17,16.94) | -6.12(-22.88,10.40) | V |

MH- exercise modality

| AT |  |  |  |  |
| --- | --- | --- | --- | --- |
| 3.95 (-1.49,10.00) | CN |  |  |  |
| -0.54(-8.90,8.16) | -4.46(-11.51,2.29) | CT |  |  |
| -2.41(-14.74,10.26) | -6.32(-19.04,6.08) | -1.83 (-15.98,12.21) | EMS |  |
| -1.66(-12.12,8.89) | -5.62(-15.18,3.57) | -1.15(-12.08,9.73) | 0.72(-14.76,16.15) | RT |

PH- exercise modality

| AT |  |  |  |  |
| --- | --- | --- | --- | --- |
| **7.26(0.33,13.87)** | CN |  |  |  |
| 2.92(-7.78,13.10) | -4.33(-13.02,4.21) | CT |  |  |
| 2.34(-13.50,17.98) | -4.95(-20.73,10.91) | -0.60 (-18.26,17.29) | EMS |  |
| -1.84(-13.98,9.63) | -9.13(-19.59,1.12) | -4.79(-17.41,7.78) | -4.13(-23.13,14.33) | RT |

# Appendix 7 Assessment of Heterogeneity results for each outcome

eTable4: Heterogeneity test result for each outcome

|  | I^2^ (Consistency) | I^2^ (Inconsistency) |
| --- | --- | --- |
| 6MWT | | |
| Exercise modality | 0 | 0 |
| Exercise intensity | 0 | 0 |
| Kt/V |  |  |
| Exercise modality | 0 | 0 |
| Exercise intensity | 0 | 0 |
| SBP |  |  |
| Exercise modality | 6 | 8 |
| Exercise intensity | 6 | 6 |
| DBP |  |  |
| Exercise modality | 9 | 8 |
| Exercise intensity | 7 | 7 |
| MH |  |  |
| Exercise modality | 6 | 6 |
| PH |  |  |
| Exercise modality | 5 | 4 |

# Appendix 8 Assessment of incoherence results for each outcome: global and local

eTable5. global incoherence for each outcome

| Model | Data points | Posterior mean deviance (Dbar) | Dbar: data points ration | Leverage (pD) | Deviance Information Criterion(DIC) |
| --- | --- | --- | --- | --- | --- |
| 6MWT | | | | | |
| Exercise modality | | | | | |
| Consistency | 42 | 32.53 | 0.77 | 25.95 | 58.48 |
| Inconsistency | 42 | 34.94 | 0.83 | 28.92 | 63.87 |
| Exercise intensity | | | | | |
| Consistency | 38 | 33.03 | 0.87 | 27.17 | 60.21 |
| Inconsistency | 38 | 32.80 | 0.86 | 28.76 | 61.57 |
| Kt/V | | | | | |
| Exercise modality | | | | | |
| Consistency | 44 | 43.02 | 0.98 | 39.86 | 82.88 |
| Inconsistency | 44 | 43.32 | 0.98 | 39.47 | 82.80 |
| Exercise intensity | | | | | |
| Consistency | 44 | 42.19 | 0.96 | 39.18 | 81.37 |
| Inconsistency | 44 | 42.16 | 0.96 | 39.15 | 81.30 |
| SBP | | | | | |
| Exercise modality | | | | | |
| Consistency | 45 | 46.67 | 1.04 | 37.08 | 83.75 |
| Inconsistency | 45 | 47.63 | 1.06 | 39.56 | 87.19 |
| Exercise intensity | | | | | |
| Consistency | 38 | 39.77 | 1.05 | 33.11 | 72.89 |
| Inconsistency | 38 | 39.48 | 1.04 | 33.61 | 73.09 |
| DBP | | | | | |
| Exercise modality | | | | | |
| Consistency | 43 | 46.36 | 1.08 | 35.45 | 81.81 |
| Inconsistency | 43 | 45.78 | 1.07 | 38.37 | 84.14 |
| Exercise intensity | | | | | |
| Consistency | 39 | 40.98 | 1.05 | 33.65 | 74.63 |
| Inconsistency | 39 | 40.74 | 1.05 | 34.10 | 74.84 |
| MH | | | | | |
| Exercise modality | | | | | |
| Consistency | 29 | 29.94 | 1.03 | 24.66 | 54.60 |
| Inconsistency | 29 | 29.87 | 1.03 | 25.61 | 55.48 |
| PH | | | | | |
| Exercise modality | | | | | |
| Consistency | 29 | 29.42 | 1.02 | 26.11 | 55.54 |
| Inconsistency | 29 | 29.15 | 1.00 | 26.91 | 56.05 |

eTable6: Evaluation of the incoherence by node-splitting model

Results of the node-splitting assessment of the evidence from direct and indirect comparisons in each outcome for different exercise modalities or intensities. The P-value for the difference is the test of the inconsistency.

| Comparison | Direct effect(Mean difference /95%CrI) | Indirect effect(Mean difference /95%CrI) | Network effect(Mean difference /95%CrI) | P-value |
| --- | --- | --- | --- | --- |
| 6MWT | | | | |
| Exercise modality | | | | |
| AT vs. CT | -1.6(-8.4,5.6) | 2.2(-1.2,5.6) | 1.3(-1.6,4.4) | 0.34 |
| AT vs. RT | -1.3(-8.8,6.6) | -0.51(-4.1,3.0) | -1.0(-4.0,2.1) | 0.85 |
| CN vs. CT | 4.9(3.1,6.7) | 0.74(-43.0,38.0) | 4.9(3.1,6.6) | 0.85 |
| CN vs. RT | 2.6(0.57,4.6) | 2.4(-40.0,45.0) | 2.5(0.64,4.5) | 1.00 |
| CT vs. RT | 0.33 (-8.3,8.2) | -2.6(-5.3,0.17) | -2.4(-4.8,0.39) | 0.54 |
| Exercise intensity | | | | |
| CN vs. L | 4.2(-0.18,8.6) | 0.96(-3.1,5.2) | 2.6(-0.44,5.4) | 0.27 |
| CN vs. M | 4.9(1.7,7.7) | 2.1(-6.8,12.0) | 4.7(1.7,7.3) | 0.56 |
| CN vs. MV | 4.5(2.0,7.1) | 9.0(3.7,14.0) | 5.4(3.0,7.7) | 0.14 |
| L vs. M | -0.11(-8.2,8.2) | 2.6(-1.7,7.0) | 2.1(-1.7,5.8) | 0.55 |
| L vs. MV | 4.6(0.72,8.5) | 0.25(-4.4,4.9) | 2.8(-0.083,5.8) | 0.16 |
| Kt/V | | | | |
| Exercise modality | | | | |
| AT vs. CT | -0.34 (-0.73, 0.05) | 0.08 (-0.22, 0.38) | -0.06 (-0.30, 0.18) | 0.09 |
| AT vs. EMS | 0.05(-0.39,0.48) | -0.16(-0.56,0.24) | -0.03(-0.31,0.27) | 0.46 |
| AT vs. RT | -0.26(-0.56,0.05) | 0.03(-0.19,0.24) | -0.07(-0.25,0.11) | 0.12 |
| CT vs. RT | -0.09(-0.40,0.23) | -0.05(-0.33,0.22) | -0.01(-0.26,0.23) | 0.84 |
| Exercise intensity | | | | |
| M vs. V | 0.01 (-0.45, 0.46) | 0.04 (-0.37, 0.44) | 0.02(-0.26, 0.29) | 0.92 |
| MV vs. V | 0.01 (-0.44, 0.46) | -0.10(-0.52,0.32) | -0.07 (-0.35, 0.23) | 0.72 |
| SBP | | | | |
| Exercise modality | | | | |
| AT vs. CT | 1.2(-29.0,32.0) | -0.92(-12.0,11.0) | -0.83(-11.0,9.5) | 0.90 |
| AT vs. EMS | 4.0(-15.0, 23.0) | 4.7(-11.0,23.0) | 5.7(-5.5,18.0) | 0.95 |
| AT vs. RT | 0.16(-29.0,30.0) | 4.1(-13.0,21.0) | 3.1(-11.0,17.0) | 0.82 |
| CN vs. RT | 0.48(-16.0,17.0) | -9.4(-32.0,15.0) | -3.1(-16.0,9.5) | 0.48 |
| CT vs. RT | -1.1 (-19.0,17.0) | 11.0(-12.0,33.0) | 3.9(-9.7,17.0) | 0.39 |
| Exercise intensity | | | | |
| CN vs. L | 6.0(-18.0,31.0) | -9.3(-34.0,14.0) | -2.1(-19.0,15.0) | 0.34 |
| CN vs. MV | -7.9(-15.0,-1.7) | 7.2(-27.0,41.0) | -7.4(-14.0,-1.3) | 0.36 |
| L vs. MV | 1.30(-22.0,25.0) | -14.0(-40.0,11.0) | -5.3(-22.0,11.0) | 0.35 |
| DBP | | | | |
| Exercise modality | | | | |
| AT vs. CT | 0.01 (-18.0, 18.0) | -0.56 (-7.5, 7.0) | -0.41 (-6.5, 6.3) | 0.96 |
| AT vs. EMS | -0.86 (-13.0, 11.0) | 5.9 (-4.1, 17.0) | 3.1 (-3.6, 10.0) | 0.35 |
| AT vs. RT | -4.0 (-23.0, 15.0) | 1.5 (-8.7, 12.0) | 0.32 ( -8.0, 8.7) | 0.61 |
| CN vs. CT | -2.7(-8.7, 3.8) | -3.3 (-21.0, 14.0) | -2.8 (-8.0, 2.8) | 0.94 |
| CN vs. RT | -1.0(-11.0, 9.0) | -2.2 (-17.0, 13.0) | -2.1(-9.8, 5.5) | 0.89 |
| CT vs. RT | -0.71(-12.0, 10.0) | 2.6 (-12.0, 17.0) | 0.73 (-7.5, 8.5) | 0.69 |
| Exercise intensity | | | | |
| CN vs. L | 6.3(-8.4, 21.0) | -3.3(-17.0, 10.0) | 1.1 (-8.9, 11.0) | 0.32 |
| CN vs. MV | -4.4 (-8.4, -0.90) | 5.0(-15.0, 25.0) | -4.1 (-7.9, -0.61) | 0.32 |
| L vs. MV | -0.96 (-14.0, 12.0) | -11.0 ( -26.0, 4.1) | -5.2 ( -15.0, 4.6) | 0.32 |
| PH | | | | |
| Exercise modality | | | | |
| AT vs. CT | 1.8(-15.0, 19.0) | 0.52 (-11.0, 12.0) | 0.54 (-8.1, 9.0) | 0.89 |
| AT vs. RT | 1.1 (-16.0, 19.0) | 4.8 (-11.0, 19.0) | 1.7 (-9.0, 12.0) | 0.73 |
| CT vs. RT | -0.77 (-17.0, 16.0) | 4.2 ( -12.0, 20.0) | 1.1 ( -9.7, 12.0) | 0.64 |
| PH | | | | |
| Exercise modality | | | | |
| AT vs. CT | -2.3 (-24.0, 19.0) | -2.7 (-17.0, 12.0) | -2.8 (-13.0, 7.7) | 0.98 |
| AT vs. RT | 1.1 (-19.0, 22.0) | 4.1 (-13.0, 22.0) | 2.0 ( -9.7, 14.0) | 0.80 |
| CT vs. RT | 3.6(-17.0, 25.0) | 6.8(-12.0,27.0) | 4.8 (-7.6, 18.0) | 0.80 |

**Appendix 9 Network analysis results for hemodialysis efficiency**

eTable7: Forest plots for Kt/V representing the comparisons of the active intervention with the control group.

| 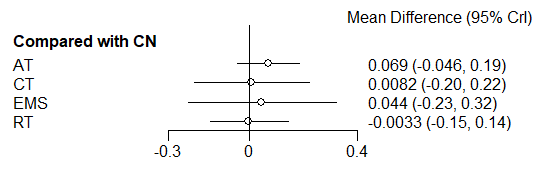 | 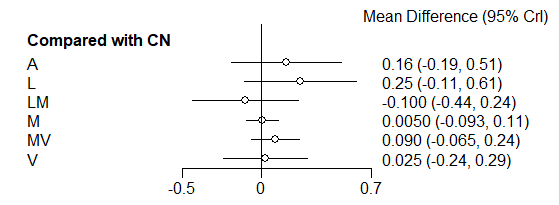 |
| --- | --- |

* Mean Difference: based on posterior distributions of the intervention relative to control; 95%CrI：95% credible intervals. AT, aerobic training; RT, resistance training; CT, combined therapy; EMS electro-stimulation; CN, control group; L, light intensity; LM, light to moderate intensity; M, moderate intensity; MV, moderate to vigorous intensity; V, vigorous intensity; A, According to patient needs.

# Appendix 10 Comparison-adjusted funnel plot for each outcome from the network meta-analysis

Funnel plots of the included studies in each outcome analysis. Each outcome was classified into three types: exercise modalities, exercise intensities, and exercise intensity plus modalities; Egger’s test was applied to test for the presence of funnel plot asymmetry.

**a. The Egger’s test Findings**

| **Outcome** | **P-value (exercise modalities)** | **P-value(exercise intensities)** |
| --- | --- | --- |
| **6MWT** | **0.02** | **0.38** |
| **KT/V** | **0.43** | **0.16** |
| **Systolic blood pressure** | **0.54** | **0.42** |
| **Diastolic blood pressure** | **0.42** | **0.44** |
| **MH** | **0.60** | **NR** |
| **PH** | **0.74** | **NR** |

**b. Comparison-adjusted funnel plot for each outcome**

| **Exercise modalities** | Exercise intensities |
| --- | --- |
| 6MWT |  |
| 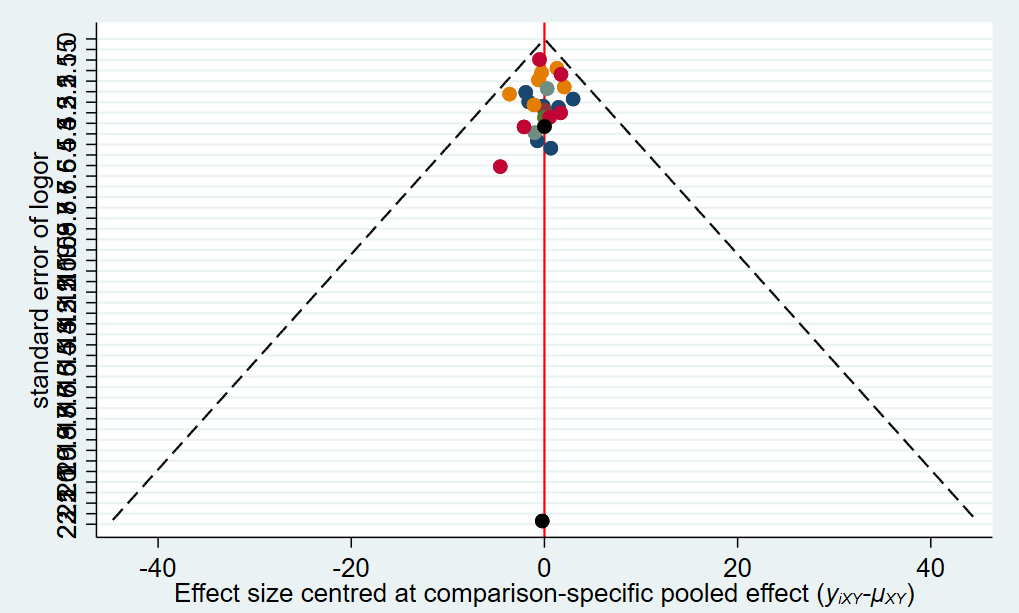 | 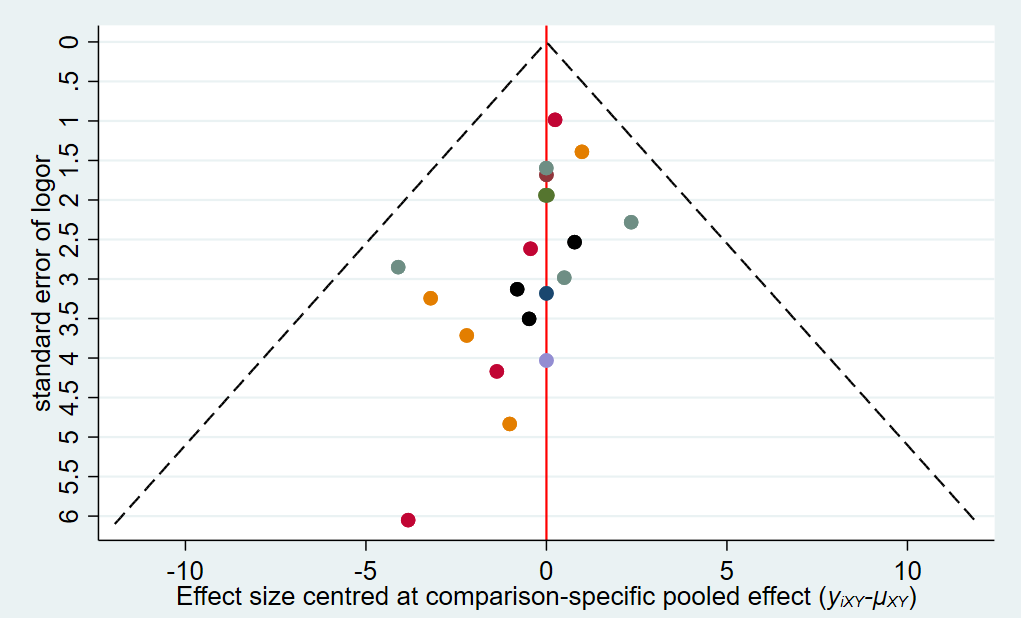 |
| KT/V |  |
| 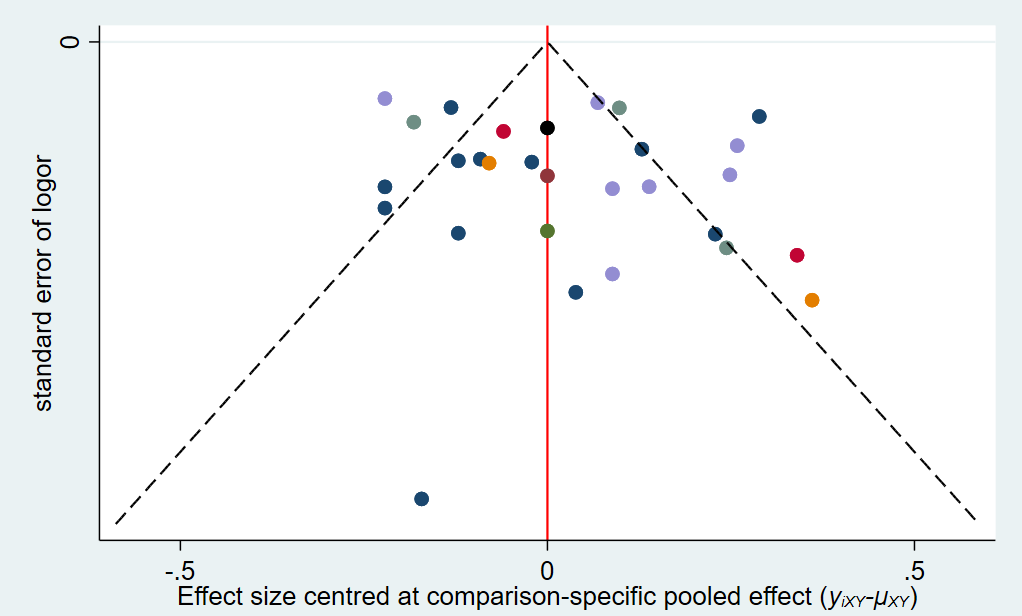 | 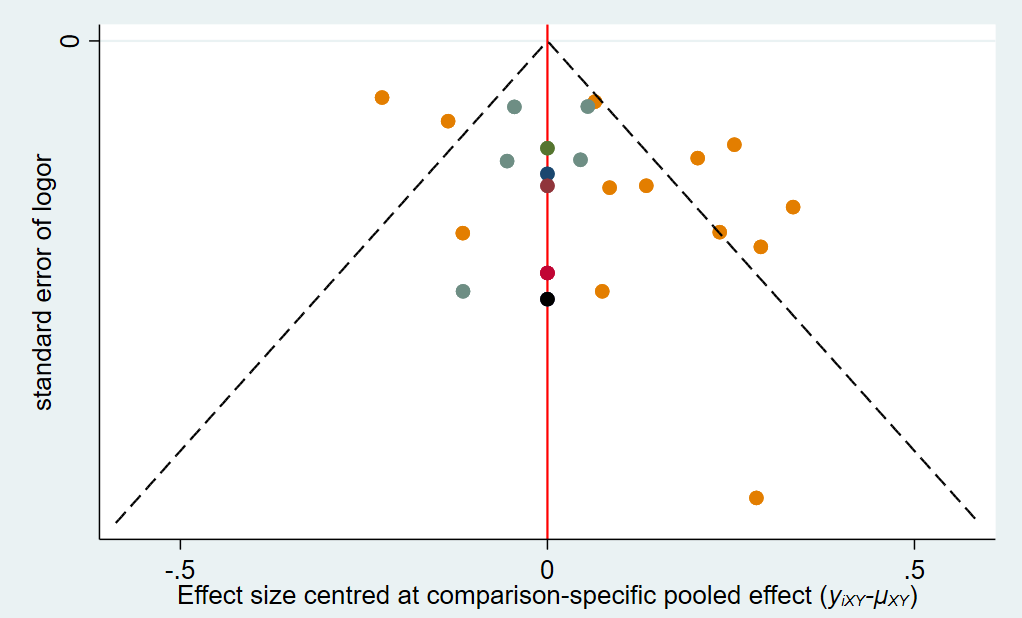 |
| **Systolic blood pressure** |  |
| 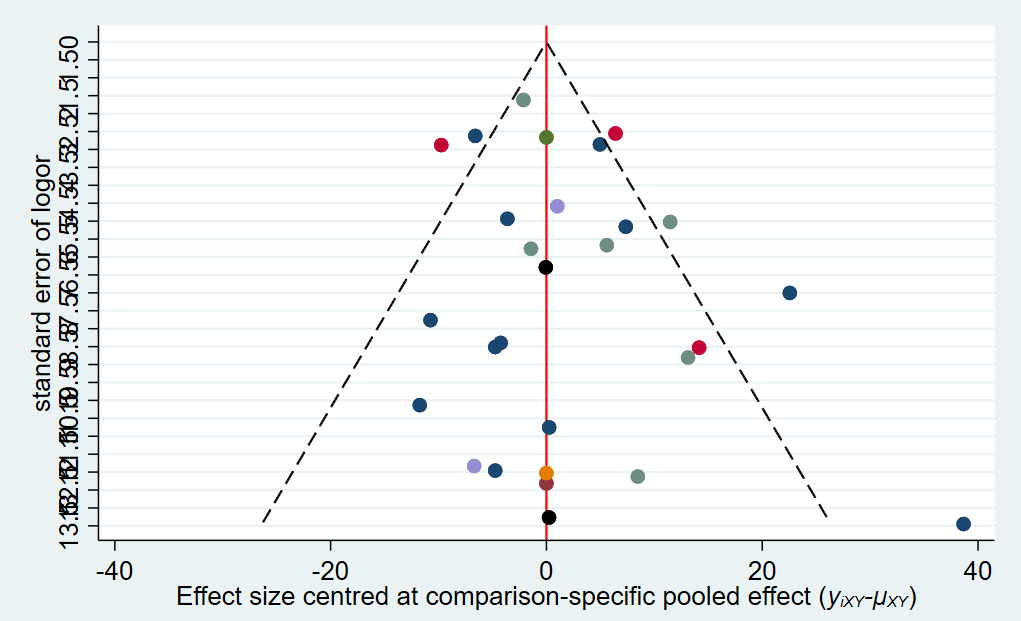 | 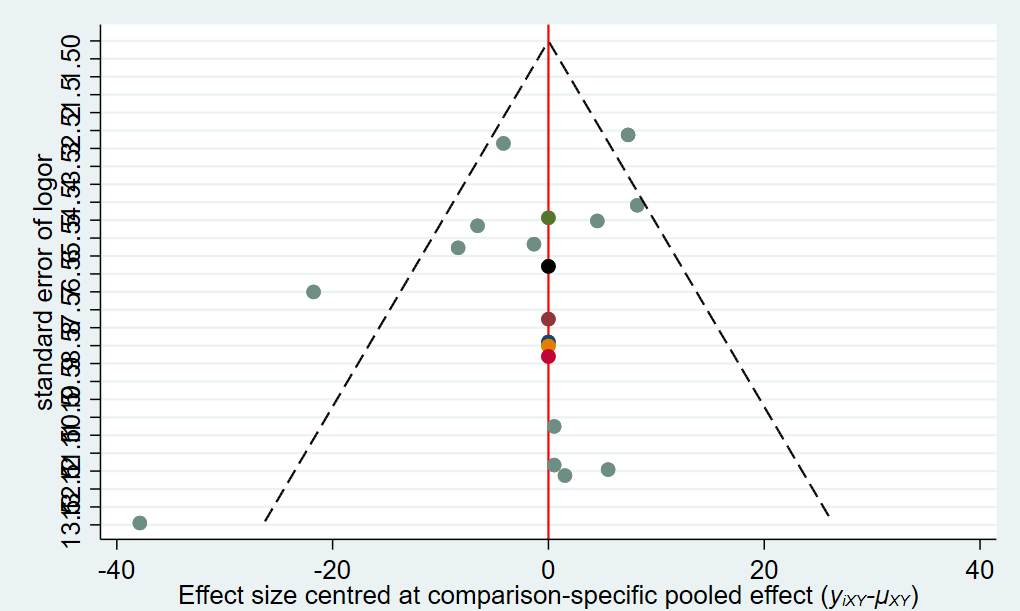 |
| **Diastolic blood pressure** |  |
| 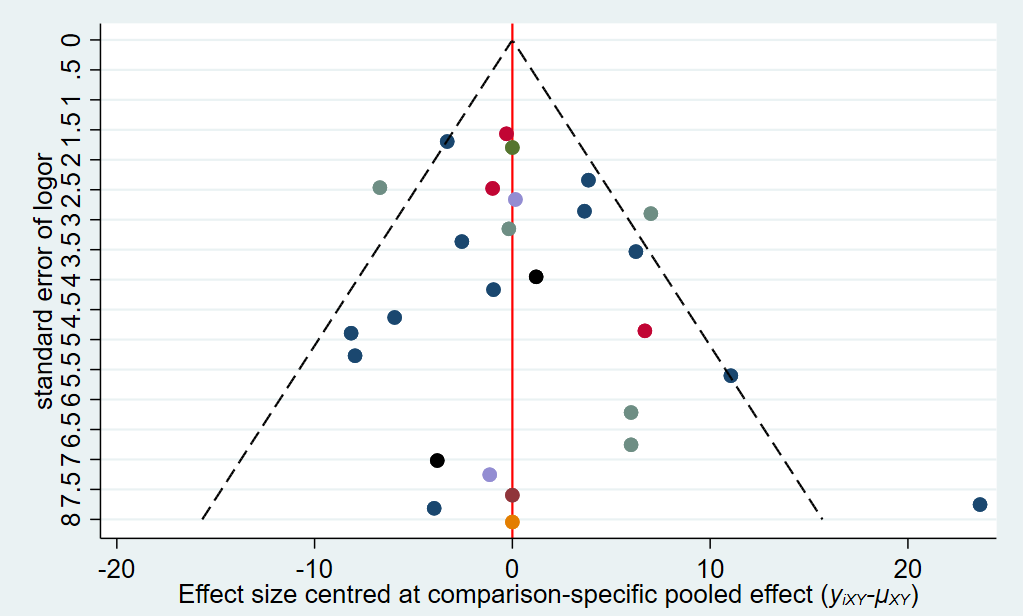 | 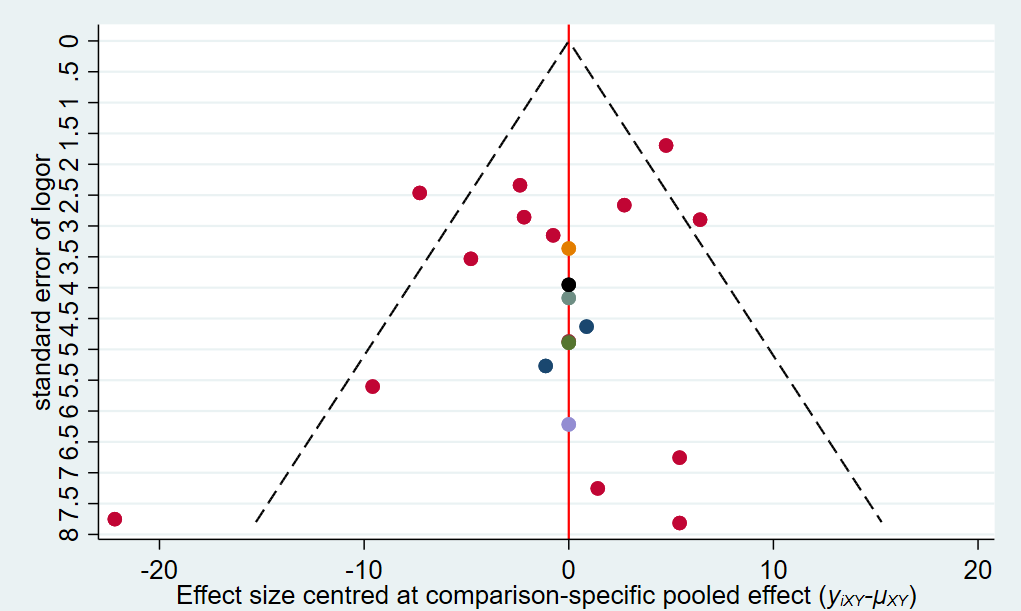 |
| **MH- Exercise modalities** | **PH- Exercise modalities** |
| 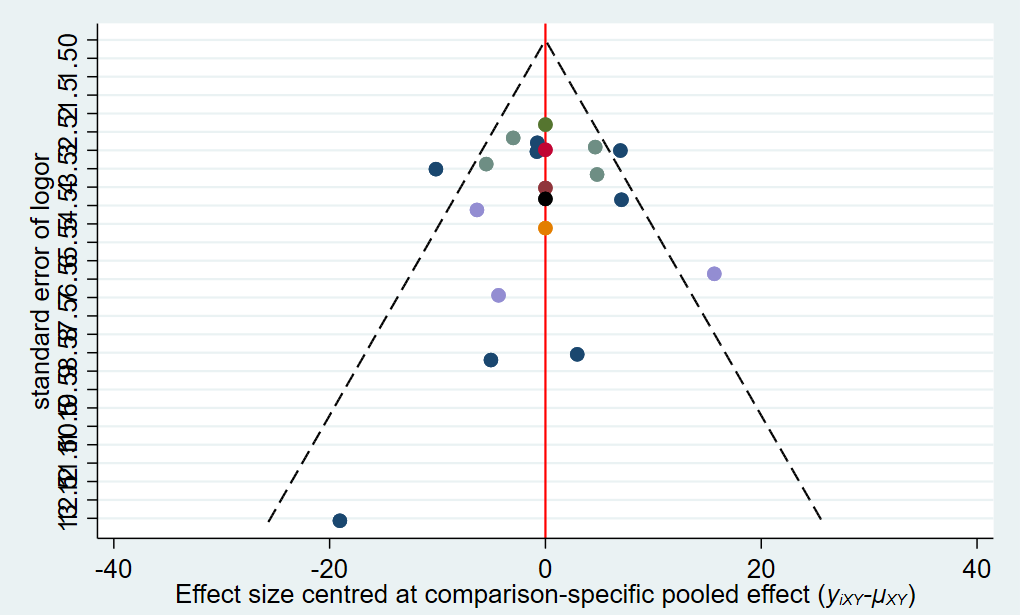 | 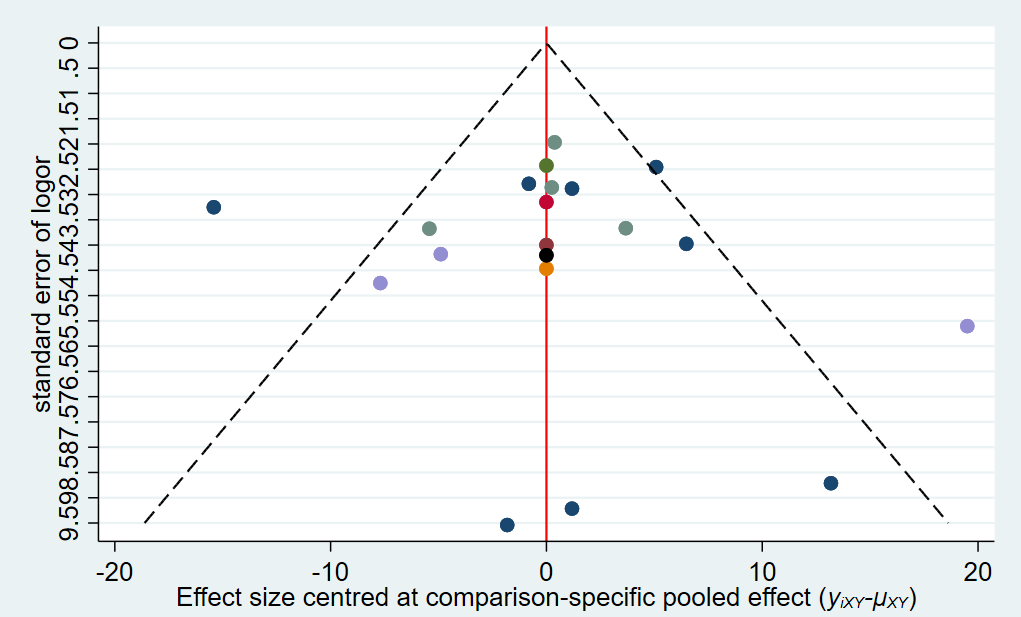 |

# Appendix 11 Network sensitivity analysis for each outcome-SURCA for each outcome

SURCA for each outcome based on exercise modality

|  | SURCA of exercise modality | | | | |
| --- | --- | --- | --- | --- | --- |
| outcome | AT | CN | CT | EMS | RT |
| 6MWT | 0.67 | 0.04 | **0.90** | 0.42 | 0.47 |
| Kt/V | **0.80** | 0.35 | 0.43 | 0.56 | 0.35 |
| SBP | 0.74 | 0.21 | **0.77** | 0.30 | 0.48 |
| DBP | 0.62 | 0.30 | **0.72** | 0.26 | 0.59 |
| MH | 0.60 | 0.18 | 0.63 | **0.76** | 0.33 |
| PH | 0.63 | 0.09 | 0.43 | 0.46 | **0.89** |

SURCA for each outcome based on exercise intensity

| SURCA of exercise intensity | | | | | | | |
| --- | --- | --- | --- | --- | --- | --- | --- |
| outcome | A | CN | L | LM | M | MV | V |
| 6MWT | 0.54 | 0.05 | 0.38 | 0.66 | 0.76 | **0.82** | 0.28 |
| Kt/V | 0.69 | 0.33 | **0.82** | 0.21 | 0.37 | 0.62 | 0.47 |
| SBP | 0.34 | 0.41 | 0.52 | 0.49 | 0.51 | **0.80** | 0.44 |
| DBP | 0.22 | 0.50 | 0.43 | 0.48 | 0.61 | **0.82** | 0.43 |

**References:**

1. Scharhag-Rosenberger F, Kuehl R, Klassen O, et al. Exercise training intensity prescription in breast cancer survivors: validity of current practice and specific recommendations. J CANCER SURVIV. 2015 2015-12-01;9(4):612-9. PMID: 25711667. DOI: 10.1007/s11764-015-0437-z.

2. Garber CE, Blissmer B, Deschenes MR, et al. American College of Sports Medicine position stand. Quantity and quality of exercise for developing and maintaining cardiorespiratory, musculoskeletal, and neuromotor fitness in apparently healthy adults: guidance for prescribing exercise. Med Sci Sports Exerc. 2011 2011-07-01;43(7):1334-59. PMID: 21694556. DOI: 10.1249/MSS.0b013e318213fefb.

3. Yoshioka M, Kosaki K, Matsui M, et al. Replacing sedentary time for physical activity on bone density in patients with chronic kidney disease. J BONE MINER METAB. 2021 2021-11-01;39(6):1091-100. PMID: 34319455. DOI: 10.1007/s00774-021-01255-w.

4. Mori S, Kosaki K, Matsui M, et al. Sedentary Behavior, Physical Activity, And Baroreflex Sensitivity In Middle-aged And Older Adults: 2683 Board #144 May 29 10:30 AM - 12:00 PM. Medicine & Science in Sports & Exercise. 2020;52(7S):738.

5. Toussaint ND, Polkinghorne KR, Kerr PG. Impact of intradialytic exercise on arterial compliance and B-type natriuretic peptide levels in hemodialysis patients. HEMODIAL INT. 2008 2008-04-01;12(2):254-63. PMID: 18394060. DOI: 10.1111/j.1542-4758.2008.00262.x.

6. Pomidori L, Lamberti N, Malagoni AM, et al. Respiratory muscle impairment in dialysis patients: can minimal dose of exercise limit the damage? A Preliminary study in a sample of patients enrolled in the EXCITE trial. J NEPHROL. 2016 2016-12-01;29(6):863-9. PMID: 27312989. DOI: 10.1007/s40620-016-0325-2.

7. Painter P, Moore G, Carlson L, et al. Effects of exercise training plus normalization of hematocrit on exercise capacity and health-related quality of life. AM J KIDNEY DIS. 2002 2002-02-01;39(2):257-65. PMID: 11840365. DOI: 10.1053/ajkd.2002.30544.

8. DePaul V, Moreland J, Eager T, Clase CM. The effectiveness of aerobic and muscle strength training in patients receiving hemodialysis and EPO: a randomized controlled trial. AM J KIDNEY DIS. 2002 2002-12-01;40(6):1219-29. PMID: 12460041. DOI: 10.1053/ajkd.2002.36887.

9. Tsuyuki K, Kimura Y, Chiashi K, et al. Oxygen uptake efficiency slope as monitoring tool for physical training in chronic hemodialysis patients. THER APHER DIAL. 2003 2003-08-01;7(4):461-7. PMID: 12887732. DOI: 10.1046/j.1526-0968.2003.00084.x.

10. Molsted S, Eidemak I, Sorensen HT, Kristensen JH. Five months of physical exercise in hemodialysis patients: effects on aerobic capacity, physical function and self-rated health. Nephron Clin Pract. 2004 2004-01-20;96(3):c76-81. PMID: 15056989. DOI: 10.1159/000076744.

11. van Vilsteren MC, de Greef MH, Huisman RM. The effects of a low-to-moderate intensity pre-conditioning exercise programme linked with exercise counselling for sedentary haemodialysis patients in The Netherlands: results of a randomized clinical trial. Nephrol Dial Transplant. 2005 2005-01-01;20(1):141-6. PMID: 15522901. DOI: 10.1093/ndt/gfh560.

12. Cheema B, Abas H, Smith B, et al. Progressive exercise for anabolism in kidney disease (PEAK): a randomized, controlled trial of resistance training during hemodialysis. J AM SOC NEPHROL. 2007 2007-05-01;18(5):1594-601. PMID: 17409306. DOI: 10.1681/ASN.2006121329.

13. Kopple JD, Wang H, Casaburi R, et al. Exercise in maintenance hemodialysis patients induces transcriptional changes in genes favoring anabolic muscle. J AM SOC NEPHROL. 2007 2007-11-01;18(11):2975-86. PMID: 17942969. DOI: 10.1681/ASN.2006070794.

14. Toussaint ND, Polkinghorne KR, Kerr PG. Impact of intradialytic exercise on arterial compliance and B-type natriuretic peptide levels in hemodialysis patients. HEMODIAL INT. 2008 2008-04-01;12(2):254-63. PMID: 18394060. DOI: 10.1111/j.1542-4758.2008.00262.x.

15. Sakkas GK, Hadjigeorgiou GM, Karatzaferi C, et al. Intradialytic aerobic exercise training ameliorates symptoms of restless legs syndrome and improves functional capacity in patients on hemodialysis: a pilot study. ASAIO J. 2008 2008-03-01;54(2):185-90. PMID: 18356653. DOI: 10.1097/MAT.0b013e3181641b07.

16. Ouzouni S, Kouidi E, Sioulis A, Grekas D, Deligiannis A. Effects of intradialytic exercise training on health-related quality of life indices in haemodialysis patients. CLIN REHABIL. 2009 2009-01-01;23(1):53-63. PMID: 19114437. DOI: 10.1177/0269215508096760.

17. Afshar R, Shegarfy L, Shavandi N, Sanavi S. Effects of aerobic exercise and resistance training on lipid profiles and inflammation status in patients on maintenance hemodialysis. Indian J Nephrol. 2010 2010-10-01;20(4):185-9. PMID: 21206679. DOI: 10.4103/0971-4065.73442.

18. Reboredo MM, Neder JA, Pinheiro BV, Henrique DM, Faria RS, Paula RB. Constant work-rate test to assess the effects of intradialytic aerobic training in mildly impaired patients with end-stage renal disease: a randomized controlled trial. Arch Phys Med Rehabil. 2011 2011-12-01;92(12):2018-24. PMID: 22133251. DOI: 10.1016/j.apmr.2011.07.190.

19. Koh KP, Fassett RG, Sharman JE, Coombes JS, Williams AD. Effect of intradialytic versus home-based aerobic exercise training on physical function and vascular parameters in hemodialysis patients: a randomized pilot study. AM J KIDNEY DIS. 2010 2010-01-01;55(1):88-99. PMID: 19932545. DOI: 10.1053/j.ajkd.2009.09.025.

20. Wilund KR, Tomayko EJ, Wu PT, et al. Intradialytic exercise training reduces oxidative stress and epicardial fat: a pilot study. Nephrol Dial Transplant. 2010 2010-08-01;25(8):2695-701. PMID: 20190243. DOI: 10.1093/ndt/gfq106.

21. Dobsak P, Homolka P, Svojanovsky J, et al. Intra-dialytic electro-stimulation of leg extensors may improve exercise tolerance and quality of life in hemodialyzed patients. ARTIF ORGANS. 2012 2012-01-01;36(1):71-8. PMID: 21848929. DOI: 10.1111/j.1525-1594.2011.01302.x.

22. Giannaki CD, Hadjigeorgiou GM, Karatzaferi C, et al. A single-blind randomized controlled trial to evaluate the effect of 6 months of progressive aerobic exercise training in patients with uraemic restless legs syndrome. Nephrol Dial Transplant. 2013 2013-11-01;28(11):2834-40. PMID: 23929523. DOI: 10.1093/ndt/gft288.

23. Mohseni R, Emami ZA, Ilali E, Adib-Hajbaghery M, Makhlough A. The effect of intradialytic aerobic exercise on dialysis efficacy in hemodialysis patients: a randomized controlled trial. Oman Med J. 2013 2013-09-01;28(5):345-9. PMID: 24044062. DOI: 10.5001/omj.2013.99.

24. Pellizzaro CO, Thome FS, Veronese FV. Effect of peripheral and respiratory muscle training on the functional capacity of hemodialysis patients. Ren Fail. 2013 2013-01-20;35(2):189-97. PMID: 23199095. DOI: 10.3109/0886022X.2012.745727.

25. Kirkman DL, Mullins P, Junglee NA, Kumwenda M, Jibani MM, Macdonald JH. Anabolic exercise in haemodialysis patients: a randomised controlled pilot study. J Cachexia Sarcopenia Muscle. 2014 2014-09-01;5(3):199-207. PMID: 24710697. DOI: 10.1007/s13539-014-0140-3.

26. Wu Y, He Q, Yin X, He Q, Cao S, Ying G. Effect of individualized exercise during maintenance haemodialysis on exercise capacity and health-related quality of life in patients with uraemia. J INT MED RES. 2014 2014-06-01;42(3):718-27. PMID: 24781720. DOI: 10.1177/0300060513509037.

27. Groussard C, Rouchon-Isnard M, Coutard C, et al. Beneficial effects of an intradialytic cycling training program in patients with end-stage kidney disease. Appl Physiol Nutr Metab. 2015 2015-06-01;40(6):550-6. PMID: 25955722. DOI: 10.1139/apnm-2014-0357.

28. Roxo RS, Xavier VB, Miorin LA, Magalhaes AO, Sens YA, Alves VL. Impact of neuromuscular electrical stimulation on functional capacity of patients with chronic kidney disease on hemodialysis. J Bras Nefrol. 2016 2016-07-01;38(3):344-50. PMID: 27737393. DOI: 10.5935/0101-2800.20160052.

29. Liao MT, Liu WC, Lin FH, et al. Intradialytic aerobic cycling exercise alleviates inflammation and improves endothelial progenitor cell count and bone density in hemodialysis patients. Medicine (Baltimore). 2016 2016-07-01;95(27):e4134. PMID: 27399127. DOI: 10.1097/MD.0000000000004134.

30. Thompson S, Klarenbach S, Molzahn A, et al. Randomised factorial mixed method pilot study of aerobic and resistance exercise in haemodialysis patients: DIALY-SIZE!. BMJ OPEN. 2016 2016-09-06;6(9):e12085. PMID: 27601500. DOI: 10.1136/bmjopen-2016-012085.

31. Frih B, Jaafar H, Mkacher W, Ben SZ, Hammami M, Frih A. The Effect of Interdialytic Combined Resistance and Aerobic Exercise Training on Health Related Outcomes in Chronic Hemodialysis Patients: The Tunisian Randomized Controlled Study. FRONT PHYSIOL. 2017 2017-01-20;8:288. PMID: 28620308. DOI: 10.3389/fphys.2017.00288.

32. Schardong J, Dipp T, Bozzeto CB, et al. Effects of Intradialytic Neuromuscular Electrical Stimulation on Strength and Muscle Architecture in Patients With Chronic Kidney Failure: Randomized Clinical Trial. ARTIF ORGANS. 2017 2017-11-01;41(11):1049-58. PMID: 28621488. DOI: 10.1111/aor.12886.

33. Abreu CC, Cardozo L, Stockler-Pinto MB, et al. Does resistance exercise performed during dialysis modulate Nrf2 and NF-kappaB in patients with chronic kidney disease? LIFE SCI. 2017 2017-11-01;188:192-7. PMID: 28887058. DOI: 10.1016/j.lfs.2017.09.007.

34. Cooke AB, Ta V, Iqbal S, et al. The Impact of Intradialytic Pedaling Exercise on Arterial Stiffness: A Pilot Randomized Controlled Trial in a Hemodialysis Population. AM J HYPERTENS. 2018 2018-03-10;31(4):458-66. PMID: 29126178. DOI: 10.1093/ajh/hpx191.

35. Rosa C, Nishimoto DY, Souza G, et al. Effect of continuous progressive resistance training during hemodialysis on body composition, physical function and quality of life in end-stage renal disease patients: a randomized controlled trial. CLIN REHABIL. 2018 2018-07-01;32(7):899-908. PMID: 29504416. DOI: 10.1177/0269215518760696.

36. McGregor G, Ennis S, Powell R, et al. Feasibility and effects of intra-dialytic low-frequency electrical muscle stimulation and cycle training: A pilot randomized controlled trial. PLOS ONE. 2018 2018-01-20;13(7):e200354. PMID: 29995947. DOI: 10.1371/journal.pone.0200354.

37. Suzuki T, Ikeda M, Minami M, et al. Beneficial Effect of Intradialytic Electrical Muscle Stimulation in Hemodialysis Patients: A Randomized Controlled Trial. ARTIF ORGANS. 2018 2018-09-01;42(9):899-910. PMID: 30069942. DOI: 10.1111/aor.13161.

38. Dong ZJ, Zhang HL, Yin LX. Effects of intradialytic resistance exercise on systemic inflammation in maintenance hemodialysis patients with sarcopenia: a randomized controlled trial. INT UROL NEPHROL. 2019 2019-08-01;51(8):1415-24. PMID: 31270740. DOI: 10.1007/s11255-019-02200-7.

39. Fernandes AO, Sens Y, Xavier VB, Miorin LA, Alves V. Functional and Respiratory Capacity of Patients with Chronic Kidney Disease Undergoing Cycle Ergometer Training during Hemodialysis Sessions: A Randomized Clinical Trial. Int J Nephrol. 2019 2019-01-20;2019:7857824. PMID: 30805216. DOI: 10.1155/2019/7857824.

40. Martins DVF, Valle PB, Almeida BA, et al. Effects of intradialytic resistance training on physical activity in daily life, muscle strength, physical capacity and quality of life in hemodialysis patients: a randomized clinical trial. DISABIL REHABIL. 2020 2020-12-01;42(25):3638-44. PMID: 31034264. DOI: 10.1080/09638288.2019.1606857.

41. Hatef M, Mousavinasab N, Esmaeili R, et al. The Effects of Exercise Training on Physical Performance and Self-efficacy in Hemodialysis Patients: A Randomized Controlled Clinical Trial. Iran J Nurs Midwifery Res. 2020 2020-11-01;25(6):520-6. PMID: 33747842. DOI: 10.4103/ijnmr.IJNMR_28_19.

42. Huang M, Lv A, Wang J, et al. Exercise Training and Outcomes in Hemodialysis Patients: Systematic Review and Meta-Analysis. AM J NEPHROL. 2019 2019-01-20;50(4):240-54. PMID: 31454822. DOI: 10.1159/000502447.

43. Yeh ML, Wang MH, Hsu CC, Liu YM. Twelve-week intradialytic cycling exercise improves physical functional performance with gain in muscle strength and endurance: a randomized controlled trial. CLIN REHABIL. 2020 2020-07-01;34(7):916-26. PMID: 32506940. DOI: 10.1177/0269215520921923.

44. Assawasaksakul N, Sirichana W, Joosri W, et al. Effects of intradialytic cycling exercise on daily physical activity, physical fitness, body composition, and clinical parameters in high-volume online hemodiafiltration patients: a pilot randomized-controlled trial. INT UROL NEPHROL. 2021 2021-02-01;53(2):359-71. PMID: 33128722. DOI: 10.1007/s11255-020-02677-7.

45. Myers J, Chan K, Chen Y, et al. Effect of a Home-Based Exercise Program on Indices of Physical Function and Quality of Life in Elderly Maintenance Hemodialysis Patients. Kidney Blood Press Res. 2021 2021-01-20;46(2):196-206. PMID: 33774634. DOI: 10.1159/000514269.

46. Savovic J, Page M, Elbers R, et al. A revised tool for assessing risk of bias in randomized trials (RoB 2.0). TRIALS. 2017;181. PMID: WOS:000410814200457.

47. Gadelha AB, Cesari M, Correa HL, et al. Effects of pre-dialysis resistance training on sarcopenia, inflammatory profile, and anemia biomarkers in older community-dwelling patients with chronic kidney disease: a randomized controlled trial. INT UROL NEPHROL. 2021 2021-10-01;53(10):2137-47. PMID: 33609277. DOI: 10.1007/s11255-021-02799-6.

48. Lin CH, Hsu YJ, Hsu PH, et al. Effects of Intradialytic Exercise on Dialytic Parameters, Health-Related Quality of Life, and Depression Status in Hemodialysis Patients: A Randomized Controlled Trial. Int J Environ Res Public Health. 2021 2021-08-31;18(17). PMID: 34501792. DOI: 10.3390/ijerph18179205.

49. Perez-Dominguez B, Casana-Granell J, Garcia-Maset R, Garcia-Testal A, Melendez-Oliva E, Segura-Orti E. Effects of exercise programs on physical function and activity levels in patients undergoing hemodialysis: a randomized controlled trial. Eur J Phys Rehabil Med. 2021 2021-12-01;57(6):994-1001. PMID: 33826277. DOI: 10.23736/S1973-9087.21.06694-6.

50. Vogiatzaki E, Michou V, Liakopoulos V, et al. The effect of a 6-month intradialytic exercise program on hemodialysis adequacy and body composition: a randomized controlled trial. INT UROL NEPHROL. 2022 2022-05-23. PMID: 35604581. DOI: 10.1007/s11255-022-03238-w.

51. Kim S, Park HJ, Yang DH. An intradialytic aerobic exercise program ameliorates frailty and improves dialysis adequacy and quality of life among hemodialysis patients: a randomized controlled trial. Kidney Res Clin Pract. 2022 2022-03-31. PMID: 35354243. DOI: 10.23876/j.krcp.21.284.

# Appendix 12 PRISMA NMA Checklist of Items to Include When Reporting A Systematic Review Involving a Network Meta-analysis

PRISMA NMA Checklist of Items to Include When Reporting A Systematic Review Involving a Network Meta-analysis

| Section/Topic | Item  # | Checklist Item | Reported on Page # |
| --- | --- | --- | --- |
| TITLE | | |  |
| Title | 1 | Identify the report as a systematic review incorporating a network meta-analysis (or related form of meta-analysis). | Page 1 |
| ABSTRACT |  | Provide a structured summary including, as applicable:  Background: main objectives  Methods: data sources; study eligibility criteria, participants, and interventions; study appraisal; and synthesis methods, such as network meta-analysis.  Results: number of studies and participants identified; summary estimates with corresponding confidence/credible intervals; treatment rankings may also be discussed. Authors may choose to summarize pairwise comparisons against a chosen treatment included in their analyses for brevity.  Discussion/Conclusions: limitations; conclusions and implications of findings.  Other: primary source of funding; systematic review registration number with registry name. | Page 1 |
| Structured | 2 |
| summary |  |
| INTRODUCTION |  |
| Rationale | 3 | Describe the rationale for the review in the context of what is already known, including mention of why a network meta- analysis has been conducted. | Page 2 |
| Objectives  METHODS | 4 | Provide an explicit statement of questions being addressed, with reference to participants, interventions, comparisons, outcomes, and study design (PICOS). | Page 3-5 |
| Protocol and registration | 5 | Indicate whether a review protocol exists and if and where it can be accessed (e.g., Web address); and, if available, provide registration information, including registration number. | Page 5 |
| Eligibility criteria | 6 | Specify study characteristics (e.g., PICOS, length of follow-up) and report characteristics (e.g., years considered, language, publication status) used as criteria for eligibility, giving rationale. Clearly describe eligible treatments included in the treatment network, and note whether any have been clustered or merged into the same node (with justification). | Page 5 |
| Information sources | 7 | Describe all information sources (e.g., databases with dates of coverage, contact with study authors to identify additional studies) in the search and date last searched. | Page 6 |
| Search | 8 | Present full electronic search strategy for at least one database, including any limits used, such that it could be repeated. | Page 6-7 |
| Study selection | 9 | State the process for selecting studies (i.e., screening, eligibility, included in systematic review, and, if applicable, | Supplement |

| included in the meta-analysis). | | |  |
| --- | --- | --- | --- |
| Data collection process | 10 | Describe method of data extraction from reports (e.g., piloted forms, independently, in duplicate) and any processes for obtaining and confirming data from investigators. | Supplement |
| Data items | 11 | List and define all variables for which data were sought (e.g., PICOS, funding sources) and any assumptions and simplifications made. | Page 4 |
| Geometry of the network | S1 | Describe methods used to explore the geometry of the  treatment network under study and potential biases related to it.  This should include how the evidence base has been graphically summarized for presentation, and what  characteristics were compiled and used to describe the evidence base to readers. | Page 4 |
| Risk of bias within individual studies | 12 | Describe methods used for assessing risk of bias of individual studies (including specification of whether this was done at the study or outcome level), and how this information is to be used in any data synthesis. | Page4 |
| Summary measures | 13 | State the principal summary measures (e.g., risk ratio, difference in means). Also describe the use of additional summary measures assessed, such as treatment rankings and surface under the cumulative ranking curve (SUCRA) values, as well as modified approaches used to present summary findings from meta-analyses. | Page5 |
| Planned methods of analysis | 14 | Describe the methods of handling data and combining results of studies for each network meta-analysis. This should include,  but not be limited to:  Handling of multi-arm trials; Selection of variance structure;  Selection of prior distributions in Bayesian analyses; and  Assessment of model fit. | Page 5 |
| Assessment of Inconsistency | S2 | Describe the statistical methods used to evaluate the agreement of direct and indirect evidence in the treatment network(s) studied. Describe efforts taken to address its presence when found. | Page 5 |
| Risk of bias across studies | 15 | Specify any assessment of risk of bias that may affect the cumulative evidence (e.g., publication bias, selective reporting within studies). | Page 5 |
| Additional analyses | 16 | Describe methods of additional analyses if done, indicating which were pre-specified. This may include, but not be limited to, the following:  Sensitivity or subgroup analyses; Meta-regression analyses;  Alternative formulations of the treatment network; and Use of alternative prior distributions for Bayesian analyses (if applicable). | Page 5 |

| 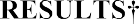 | | |  |
| --- | --- | --- | --- |
| Study selection | 17 | Give numbers of studies screened, assessed for eligibility, and included in the review, with reasons for exclusions at each stage, ideally with a flow diagram. | Page 6 |
| Presentation of network structure | S3 | Provide a network graph of the included studies to enable visualization of the geometry of the treatment network. | Page 6 |
| Summary of network geometry | S4 | Provide a brief overview of characteristics of the treatment network. This may include commentary on the abundance of trials and randomized patients for the different interventions and pairwise comparisons in the network, gaps of evidence in the treatment network, and potential biases reflected by the network structure. | Supplement |
| Study characteristics | 18 | For each study, present characteristics for which data were extracted (e.g., study size, PICOS, follow-up period) and provide the citations. | Supplement |
| Risk of bias within studies | 19 | Present data on risk of bias of each study and, if available, any outcome level assessment. | Page 8-9 |
| Results of individual studies | 20 | For all outcomes considered (benefits or harms), present, for each study: 1) simple summary data for each intervention group, and 2) effect estimates and confidence intervals.  Modified approaches may be needed to deal with information from larger networks. | Page 10 |
| Synthesis of results | 21 | Present results of each meta-analysis done, including confidence/credible intervals. In larger networks, authors may focus on comparisons versus a particular comparator (e.g. placebo or standard care), with full findings presented in an appendix. League tables and forest plots may be considered to summarize pairwise comparisons. If additional summary measures were explored (such as treatment rankings), these should also be presented. | Page 11-13 |
| Exploration for inconsistency | S5 | Describe results from investigations of inconsistency. This may include such information as measures of model fit to compare consistency and inconsistency models, P values from statistical tests, or summary of inconsistency estimates from different parts of the treatment network. | Page 8-9 |
| Risk of bias across studies | 22 | Present results of any assessment of risk of bias across studies for the evidence base being studied. | Supplement |
| Results of additional analyses  DISCUSSION | 23 | Give results of additional analyses, if done (e.g., sensitivity or subgroup analyses, meta-regression analyses, alternative network geometries studied, alternative choice of prior distributions for Bayesian analyses, and so forth). | Page 13 |
| Summary of evidence | 24 | Summarize the main findings, including the strength of evidence for each main outcome; consider their relevance to key groups (e.g., healthcare providers, users, and policy- makers). | Page 14-15 |
| Limitations | 25 | Discuss limitations at study and outcome level (e.g., risk of bias), and at review level (e.g., incomplete retrieval of identified research, reporting bias). Comment on the validity of the assumptions, such as transitivity and consistency. Comment | Page 18 |

| on any concerns regarding network geometry (e.g., avoidance of certain comparisons). | | |  |
| --- | --- | --- | --- |
| Conclusions | 26 | Provide a general interpretation of the results in the context of other evidence, and implications for future research. | Page 19 |
| FUNDING  Funding | 27 | Describe sources of funding for the systematic review and other support (e.g., supply of data); role of funders for the systematic review. This should also include information regarding whether funding has been received from manufacturers of treatments in the network and/or whether some of the authors are content experts with professional conflicts of interest that could affect use of treatments in the network. | Page 19 |

PICOS = population, intervention, comparators, outcomes, study design.

* Text in italics indicates wording specific to reporting of network meta-analyses that has been added to guidance from the PRISMA statement.

Authors may wish to plan for use of appendices to present all relevant information in full detail for items in this section.
